# Loss of angiopoietin-2 leads to region-specific brain malformations and blood-brain barrier leakage

**DOI:** 10.1172/jci.insight.198256

**Published:** 2026-02-19

**Authors:** Weihan Li, Elisa Vázquez-Liébanas, Chanaëlle Fébrissy, Florent Sauvé, Jianhao Wang, Doğan E. Sayıner, Pia Buslaps, Amanda Norrén, Michael Vanlandewijck, Liqun He, Marie Jeansson, Lars Muhl, Maarja Andaloussi Mäe

**Affiliations:** 1 Department of Immunology, Genetics, and Pathology, Rudbeck Laboratory, Uppsala University, Uppsala, Sweden.; 2 Single Cell Core Facility of Flemingsberg Campus (SICOF), Karolinska Institute, Huddinge, Sweden.; 3 Department of Medicine, Huddinge, Karolinska Institute, Huddinge, Sweden.; 4 Department of Clinical Medicine, University of Bergen, Bergen, Norway.

**Keywords:** Cell biology, Vascular biology, Angiogenesis, Endothelial cells, Transcriptomics

## Abstract

Angiopoietin-2 (ANGPT2) is known to destabilize vascular barriers in most peripheral organs; however, its role in the brain vasculature remains poorly understood. To investigate its physiological function within the brain vasculature, we analyzed constitutive *Angpt2*-knockout mice in adulthood. We showed that loss of ANGPT2 leads to region-specific vascular malformations and blood-brain barrier (BBB) dysfunction, resulting in differential permeability to 1 kDa and 70 kDa fluorescent tracers. Notably, overt vascular malformations appeared only in select brain regions that allowed leakage of both tracers. These malformations were characterized by dilated, intertwined, and sprouting endothelial cells, surrounded by reactive perivascular cells, along with high levels of astrocyte- and neuron-derived vascular endothelial growth factor A (VEGFA) and elevated expression of the vascular receptors VEGF receptor 2 (KDR) and neuropilin-1 (NRP1). Other cortical areas without obvious malformations exhibited significant leakage of the 1 kDa tracer. We also demonstrated that different cell types took up the tracers after passing the BBB. Our findings identified ANGPT2 as an important factor involved in the regulation of cerebrovascular architecture, barrier integrity, and endothelial-parenchymal interactions, and uncovered surprising differences in the leakage patterns and cellular uptake of two widely used BBB tracers.

## Abstract 2

Our work investigates the role of Angiopoietin 2 (ANGPT2) in brain vasculature, where its function remains largely undefined despite its known impact on vascular destabilization in peripheral organs. We found that ANGPT2, typically undetectable in the healthy adult brain but elevated in certain disease states, like human stroke and glioblastoma, plays a critical role in maintaining cerebrovascular integrity. Loss of *Angpt2* led to region-specific vascular malformations and blood-brain barrier (BBB) dysfunction, with differential permeability to 1 and 70 kDa fluorescent tracers. Some areas exhibited pronounced vascular malformations characterized by leaky, dilated, and sprouting vessels with distinct gene expression profiles and a reactive cellular environment in the parenchyma, while other regions showed selective leakage of smaller tracer despite appearing morphologically normal. Notably, we also discovered that tracer uptake post-BBB breach is cell type-specific. These findings reveal ANGPT2 as a key regulator of cerebrovascular architecture and barrier function, uncovering a novel dichotomy in tracer permeability and parenchymal response that has broad implications for understanding BBB dynamics in health and disease.

## Graphical abstract

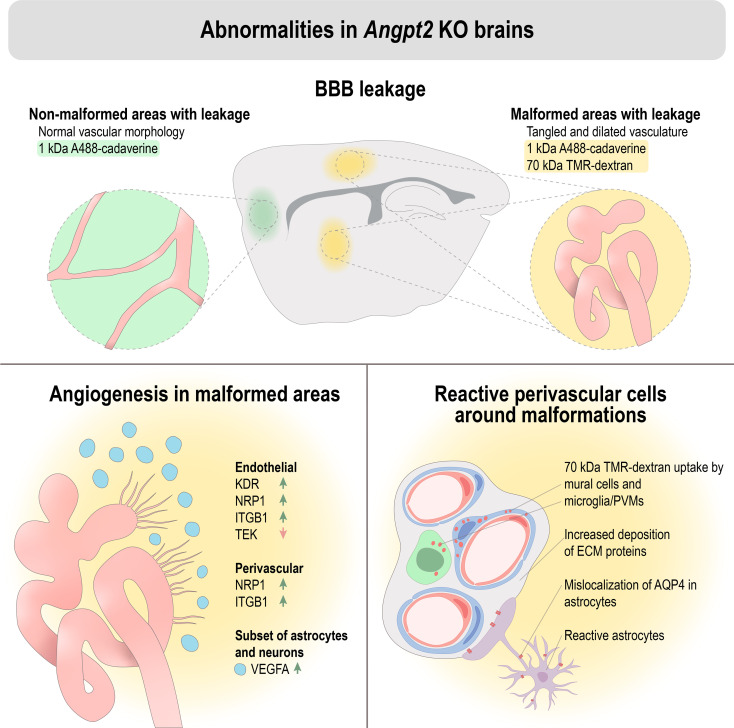

## Introduction

The blood-brain barrier (BBB) is formed by brain vascular endothelial cells. Its integrity is essential for maintaining a stable extracellular milieu to support neuronal homeostasis; however, it also prevents drug delivery to the brain for treatment of brain diseases. BBB disruption has been reported in several brain disorders (1), but the consequences for disease pathogenesis, as well as the molecular and cellular mechanisms involved, remain incompletely understood.

Upregulation of angiopoietin-2 (ANGPT2) expression is a common hallmark of vascular reactivity to disease or other challenges. Angiopoietin signaling in different organs in health and disease has been a major focus in vascular biology over the past 25 years. There are two main angiopoietin ligands, ANGPT1 and ANGPT2, that signal through the TEK receptor tyrosine kinase (TEK). The orphan receptor tyrosine kinase with immunoglobulin-like and EGF-like domains 1 (TIE1) is known to affect the response of TEK (2). ANGPT1 acts as a TEK agonist important for vascular remodeling, maturation, and stabilization (3, 4). Unlike ANGPT1, ANGPT2 is a context-dependent TEK agonist or antagonist (5–7) and can also signal via integrins independently of TEK or TIE1 (8). In many peripheral organs, ANGPT2 destabilizes endothelial barriers by antagonizing signaling through TEK receptors (9, 10). Very little, however, is known about the role of ANGPT2 in the brain. We have previously found that ANGPT2 expression is elevated in the brain vasculature of mouse models with BBB permeability caused by congenital or adult-induced pericyte hypoplasia, or by adult-onset opening of the endothelial tight junctions through induced deletion of the claudin-5 (*Cldn5*) gene (11–13). Several other mouse models have demonstrated elevated *Angpt2* expression, including endothelial cell–specific knockout (KO) of *Smad4*, *Alk1*, or *Eng* genes, which are all models for hereditary hemorrhagic telangiectasia (14). Increased *Angpt2* expression has also been documented in humans in glioblastoma (15, 16) and in stroke (17).

In adult mice with congenital pericyte hypoplasia (*Pdgfb^ret/ret^* mice), strong ANGPT2 expression was found in brain endothelium devoid of pericyte contact, while negligible levels of ANGPT2 were found in vessels with residual pericyte contact similar to the situation in controls, suggesting paracrine regulation of ANGPT2 expression (11). Based on these findings we hypothesized that ANGPT2 might be a driver of BBB leakage in vessels without pericytes, as well as in the other above-mentioned conditions of ANGPT2 overexpression and BBB breakdown. However, contrary to our assumption, deletion of *Angpt2* in *Pdgfb^ret/ret^* mice led to a further increase in BBB permeability, indicating that ANGPT2 exerts a protective or reinforcing, rather than destabilizing, role at the BBB. These findings emphasize the complex and context-dependent nature of ANGPT2 signaling and underscore the need for systematic investigation of its diverse functions at the BBB.

We previously discovered that constitutive *Angpt2* deletion in mice leads to vascular abnormalities and BBB leakage in the adult brain (11). In the present study, we provide a deeper mechanistic insight into the vascular abnormalities in *Angpt2*-KO mice. We demonstrate that ANGPT2 is essential for region-specific development and function of the cerebrovasculature. Constitutive loss of *Angpt2* leads to the formation of vascular malformations primarily in the somatosensory cortex (SS) layers 4–6 (SS4–6) and the caudoputamen (CP) in adult mouse brains. These malformed regions exhibit increased BBB permeability to fluorescent tracers of 1 and 70 kDa, angiogenic sprouting without concomitant endothelial proliferation, and elevated extracellular matrix (ECM) deposition. Interestingly, other cortical regions — including the agranular insular (AI), orbital (ORB), and somatomotor (MO) areas — showed selective leakage of the 1 kDa tracer but not the 70 kDa tracer, despite macroscopically normal vascular morphology, thereby identifying different modes of BBB dysfunction in *Angpt2*-KO mice. We show that the region-specific morphological changes in mural cells, microglia, and astrocytes correlate with the type and extent of BBB disruption. Single-cell RNA sequencing (scRNA-seq) analyses provide further information about the angiogenic phenotype by identifying differentially expressed genes associated with angiogenesis. Notably, within the malformed regions, vascular endothelial growth factor A (VEGFA) was upregulated in a subset of astrocytes and neurons, and its receptor VEGFR2 (KDR) and coreceptor neuropilin-1 (NRP1) were both upregulated in the malformed vasculature. Collectively, our data demonstrate that ANGPT2 is essential for cerebrovascular development and BBB integrity, as its constitutive loss leads to region-specific vascular malformations with aberrant angiogenic signaling, and differential BBB disruption through coordinated changes in endothelial cells, mural cells, glia, and neurons.

## Results

### Angpt2-KO mice develop vascular malformations in the CP and SS4–6.

*Angpt2*-KO mice are adult viable but display a few residual consequences of developmental lymphatic and retinal vascular defects (5). We recently discovered that *Angpt2*-KO mice also exhibit abnormal brain vascular morphology and increased leakage of the 1 kDa cadaverine in the brain (11), which prompted us to systematically analyze the brain vasculature in adult *Angpt2*-KO mice.

**Figure 1:**
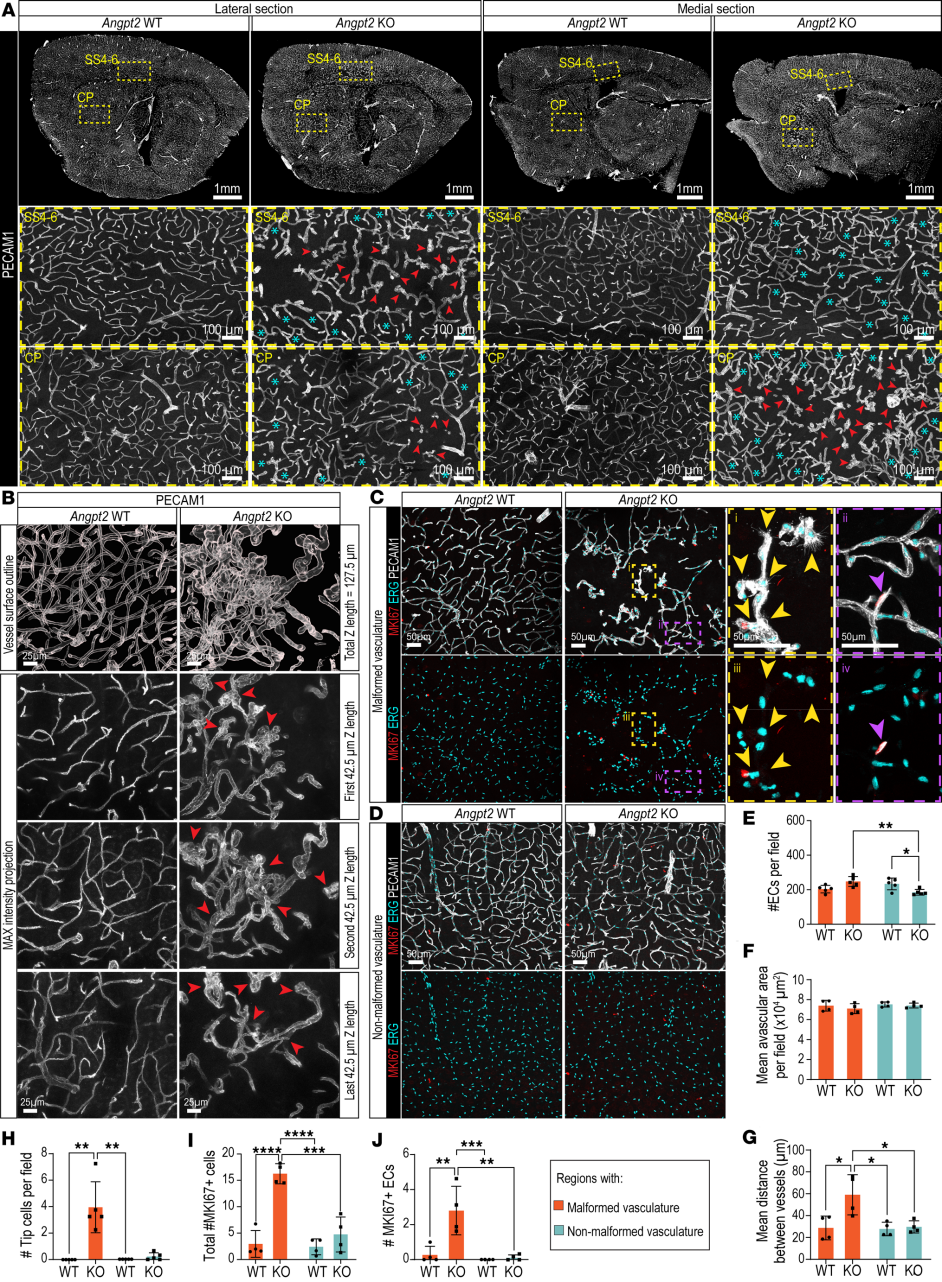
Vascular malformations and vascular sprouting in adult *Angpt2*-KO brains. (**A**) Representative tile scans of PECAM1 (gray) IF on sagittal sections of *Angpt2* WT (*n* = 24) and KO (*n* = 25). High-magnification images of SS4–6 and CP are shown. Ball-like abnormal vasculature (red arrowheads); dilated vessels surrounding ball-like structures (cyan asterisk). (**B**) IF of PECAM1 (gray) in CP of cleared 250-μm-thick brain sections (*n* = 2). Bulged and intertwined vessels (red arrowheads). Scale bars: 25 μm. (**C**) Representative images of ERG (cyan), PECAM1 (gray), and MKI67 (red) IF in CP (*n* = 4). Tip cells (yellow arrowheads) and proliferative ECs (purple arrowheads) CP are shown at higher magnification below. Scale bars: 50 μm. (**D**) Representative images of ERG (cyan), PECAM1 (gray), and MKI67 (red) IF in SS1–3 (*n* = 4). Scale bars: 50 μm. (**E**–**J**) Quantifications of malformed (orange bars) and non-malformed vasculature (light blue bars). (**E**) Quantification of EC number per field (*n* = 5). (**F**) Quantification of mean avascular area per field (*n* = 4). (**G**) Quantification of mean distance between vessels (*n* = 4). (**H**) Quantification of the number of tip cells per field (*n* = 5). (**I**) Quantification of the total number of MKI67^+^ cells per field (*n* = 4). (**J**) Quantification of the number of MKI67^+^ ECs per field (*n* = 4). (**H**) Data were not normally distributed, and a Kruskal-Wallis test was used to test significance. **P < 0.01. (**E**–**G**, **I**, and **J**) Data were normally distributed, and a 1-way ANOVA with Tukey’s multiple-comparison test was performed to evaluate significance. *P < 0.05, **P < 0.01, ***P < 0.001, ****P < 0.0001. Scale bars: 100 μm (**A**); 50 μm (**C**).

To get a detailed view of vascular anatomy across different brain regions, we performed immunofluorescent (IF) staining of the pan–endothelial cell (pan-EC) marker PECAM1 on brain sections covering the entire cerebrum in 2- to 3-month-old *Angpt2*-KO mice and littermate controls. This analysis revealed morphological vascular abnormalities consistently in two brain regions of the *Angpt2*-KO mice, CP and SS4–6 (Figure 1A and Supplemental Figure 1A; supplemental material available online with this article; https://doi.org/10.1172/jci.insight.198256DS1), and less consistently also in other brain regions, including the caudal part of the MO cortex, thalamus, and nucleus accumbens (Supplemental Figure 1B). Therefore, we concentrated our further analysis of the vascular malformation to lateral and medial sections with visible CP and SS4–6. The core of these abnormalities in *Angpt2*-KO brains displayed irregular intertwined ball-like vascular structures that were surrounded by a dilated vascular network, which gradually transitioned into macroscopically normal-looking vessels (Figure 1A). We repeatedly found a normal vascular morphology in SS1, the visual cortex (VIS), and the rostral part of the MO cortex throughout the studied *Angpt2*-KO brains. Furthermore, the gradual transition from abnormal to normal vasculature morphology made it challenging to define a clear boundary of the abnormal regions, and therefore, all quantifications made outside of the malformations are referred to as regions with “non-malformed vasculature.” To achieve a detailed understanding of the 3D architecture of the malformed vasculature, we cleared PECAM1-stained 250-μm-thick brain sections and imaged with confocal microscopy. This analysis showed that the vessels within the malformations were extensively tangled and abnormally dilated, occasionally bulging into microaneurysm-like structures (Figure 1B and Supplemental Videos 1 and 2).

To further investigate the cellular organization and proliferative characteristics of malformed and non-malformed vasculature in *Angpt2*-KO brains, we compared IF stainings for PECAM1, the general cell cycle marker MKI67, and the EC-specific transcription factor and nuclear marker ERG, in corresponding regions in *Angpt2*-KO and WT controls (Figure 1, C–J). This showed that in the malformed regions of *Angpt2*-KO, the mean number of ECs per microscopic field and avascular areas remained similar (Figure 1, E and F); however, the mean distance between the vessels was significantly increased when compared with the corresponding regions in WT (Figure 1G). In non-malformed regions, the mean EC number per vascular area was reduced in KO (Figure 1E); however, the mean avascular area and mean distance between vessels were comparable between *Angpt2* WT and KO (Figure 1, F and G).

The malformed vessels displayed signs of active angiogenic sprouting. They carried abundant endothelial tip cells, as evident by the morphology of blunt-ended sprouts with numerous protruding filopodia (Figure 1C). The presence of angiogenic sprouts and tip cells was largely limited to the regions with malformed vasculature in *Angpt2*-KO brains; only rare tip cells could be found in the regions outside malformations in *Angpt2*-KO brains, and none could be identified in *Angpt2*-WT brains (Figure 1, C and H). Higher numbers of proliferating (MKI67^+^) cells were observed in the *Angpt2*-KO regions with malformed vasculature compared with regions with non-malformed vasculature and comparable areas in WT controls (Figure 1, C and I). However, most (≈80%) of the proliferating cells in malformed regions were non-endothelial (Figure 1, C, I, and J). EC proliferation was very low in regions with non-malformed vasculature and in WT controls (Figure 1, C and J).

### Angpt2-KO mice display distinct regional modes of BBB permeability.

When addressing the nature of the leakage discovered in *Angpt2*-KO mice (11), we considered that our previous work revealed different consequences for the molecular type and size selectivity of tracer leakage across the BBB depending on whether its tight junctions had been disrupted (12), or whether endothelial transcytosis had been activated due to pericyte deficiency (11, 18).

**Figure 2:**
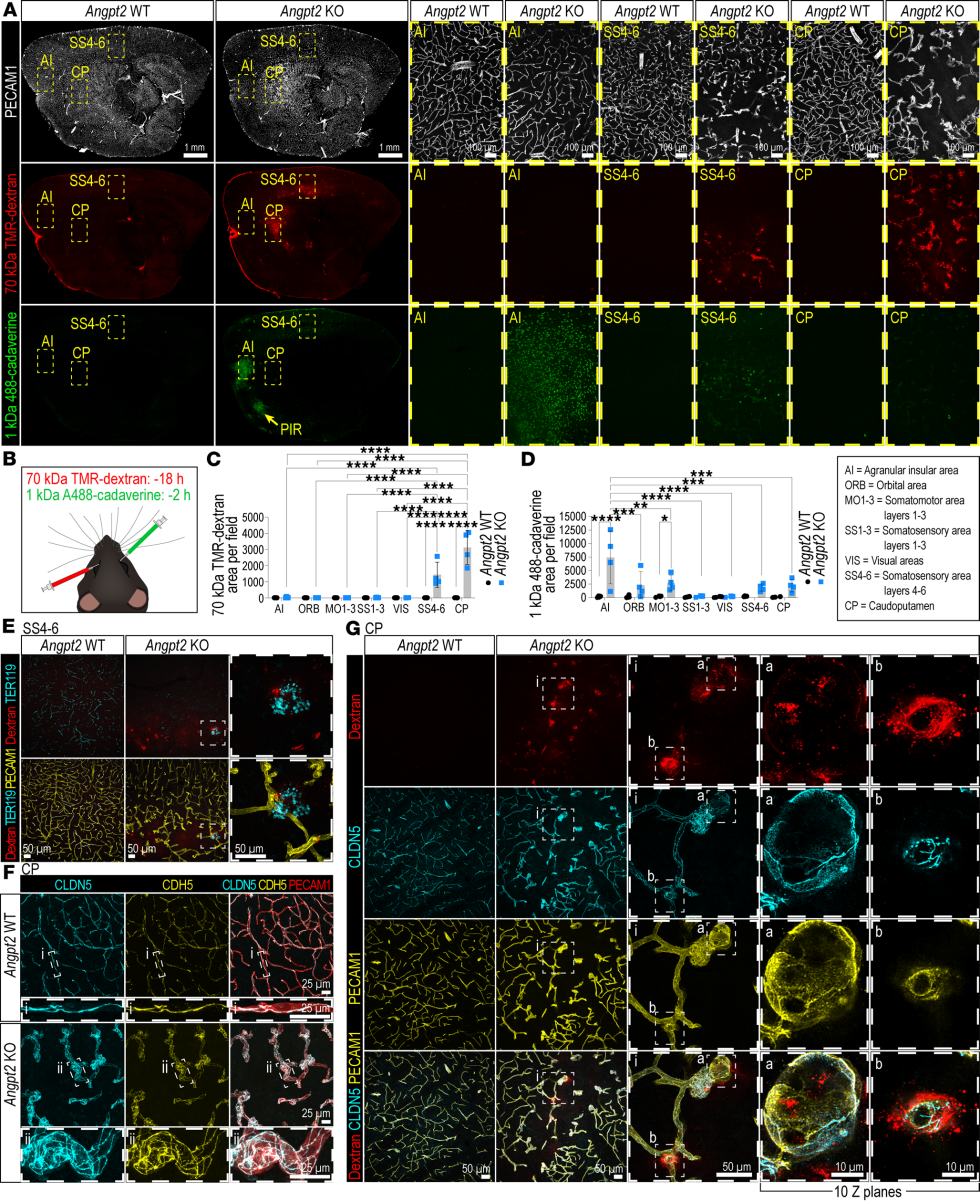
BBB permeability and endothelial junctions in *Angpt2-*KO brains. (**A**) Tile scans of PECAM1 (gray) IF with 70 kDa TMR-dextran (red) and 1 kDa A488-cadaverine (green) on sagittal sections (*n* = 4). High-magnification images of different modes of leakage in malformed (SS4–6, CP) and non-malformed vasculature (AI) are shown. Yellow arrow points to piriform area (PIR) with occasional A488-cadaverine leakage. (**B**) Illustration of intravenous retro-orbital approach used for tracer injection. (**C** and **D**) Quantification of 70 kDa TMR-dextran (**C**) and 1 kDa A488-cadaverine (**D**) leakage in different cortical regions (AI, ORB, MO, SS1–3, SS4–6, and VIS) and CP (*n* = 4). Data were normally distributed, and a 2-way ANOVA with Tukey’s multiple-comparison test was performed to evaluate significance. *P < 0.05, **P < 0.01, ***P < 0.001, ****P < 0.0001. (**E**) Representative images of PECAM1 (yellow) and TER119 (cyan) IF and 70 kDa TMR-dextran (red) in SS4–6 from *Angpt2* WT (*n* = 5) and KO (*n* = 6). High-magnification images of red blood cell extravasation in malformed vasculature are shown. (**F**) Representative images of PECAM1 (red), CLDN5 (cyan), and CDH5 (yellow) IF in CP from *Angpt2* WT (*n* = 5) and KO (*n* = 6). High-magnification images of a regular vascular stretch in *Angpt2* WT and a dilated and tangled vascular stretch in *Angpt2* KO are shown. Scale bars: 25 μm. (**G**) Representative images of PECAM1 (yellow) and CLDN5 (cyan) IF and 70 kDa TMR-dextran (red) in CP (*n* = 4). High-magnification images of parenchymal dextran leakage in malformed vasculature are shown. Scale bars: 100 μm (**A**); 50 μm (**E**); 25 μm (**F**); 50 and 10 μm (**G**).

To further investigate how *Angpt2* deficiency impacts the BBB, we sequentially injected 2 fluorescent tracers of different sizes intravenously in each mouse. First, 70 kDa tetramethylrhodamine-dextran (TMR-dextran) was injected; this tracer is known to leak across the BBB in pericyte-deficient mice with increased BBB transcytosis (11, 18) but not in mice with deficient endothelial tight junctions at the BBB (12). Sixteen hours later (2 hours before tissue collection), 1 kDa Alexa Fluor 488 cadaverine (A488-cadaverine) was injected (Figure 2, A and B). We found 70 kDa TMR-dextran extravasation only around the malformed vasculature (in SS4–6 and CP) of *Angpt2* KO (Figure 2, A and C). The non-malformed vasculature in *Angpt2* KO, and all corresponding regions in *Angpt2*-WT brains, lacked signs of TMR-dextran extravasation (Figure 2, A and C). In contrast, extravasation of A488-cadaverine was detected both in regions with malformed vessels and in certain regions with non-malformed vasculature (AI, ORB, and MO layers 1–3) (Figure 2, A and D). We also detected inconsistent A488-cadaverine leakage in the piriform area in some mice (Figure 2A). Other regions with non-malformed vessels in *Angpt2* KO (SS1–3 and VIS) lacked signs of A488-cadaverine extravasation (Figure 2, A and D). A488-cadaverine extravasation, like TMR-dextran extravasation, was not detected in any regions in WT mice. As we have previously noted (18), fluorescently labeled 1 kDa cadaverine is rapidly taken up by parenchymal cells (Figure 2A). We found that the majority of the A488-cadaverine–positive cells were neurons, as judged by costaining for the pan-neuronal marker NEUN (positive) and the pan-oligodendrocyte progenitor marker OLIG2 (negative) (12, 18). A minor proportion of the A488-cadaverine–positive cells were negative for both NEUN and OLIG2, suggesting that other cells also may take up A488-cadaverine (Supplemental Figure 2, A–C). Differences in the A488-cadaverine–positive neurons’ NEUN intensity and regional distribution were noted (Supplemental Figure 2, A and B), though the underlying reasons for this remain unclear. Notably, the cellular uptake of A488-cadaverine was stronger in the leaky non-malformed than in the malformed regions in *Angpt2* KO (Figure 2, A and D), perhaps suggesting that the parenchymal cells are functionally impaired (with regard to tracer uptake) in the malformed regions. IF staining with a TER119 antibody revealed focal hemorrhages around some of the malformed vessels in the *Angpt2* KO. In contrast, no hemorrhages were observed in regions with non-malformed vasculature in the *Angpt2* KO or in WT controls (Figure 2E).

Because our previous work indicated potential changes in the expression of CLDN5 in *Angpt2*-KO brains (11), we characterized endothelial junctional morphology across malformed and non-malformed regions in *Angpt2*-KO mice using IF staining of the tight junction proteins CLDN5 and zonula occludens 1 (ZO1) and the adherens junction proteins cadherin 5 (CDH5) and PECAM1 (12). Overall, the junctional patterns in the regions with malformed vasculature were irregular and clearly distinguishable from the patterns in surrounding regions with dilated and non-malformed vasculature in *Angpt2*-KO mice and corresponding areas in WT controls (Figure 2, F and G, and Supplemental Figure 2D). However, we concluded that these features likely reflect the tangled and dilated characteristics of the vessels in regions with malformed vasculature, as the distribution of CLDN5/ZO1 and CDH5 was continuous along endothelial junctions throughout the regions with malformed vasculature (Figure 2F and Supplemental Figure 2D). Notably, we did not find CDH5^+^ junctions that were negative for CLDN5, as previously reported (11), which likely reflects the improved IF staining protocols used in the present study. However, we need to be careful with our interpretations of these observations, as the junctional arrangements in the malformed blood vessels (with extensive TMR-dextran leakage) are complicated to assess owing to the tangled vascular morphology at these sites (Figure 2F).

### Angpt2-KO mice display region-specific changes in mural cells and fibroblasts.

Given the proposed role of Angpt/Tie signaling in endothelial–mural cell interactions (2), we examined perivascular cells in regions exhibiting different types of vascular leakage. We performed IF staining for a panel of mural cell markers and compared their distribution in leaky versus non-leaky regions in *Angpt2*-KO mice, as well as in the corresponding regions of WT controls.

**Figure 3:**
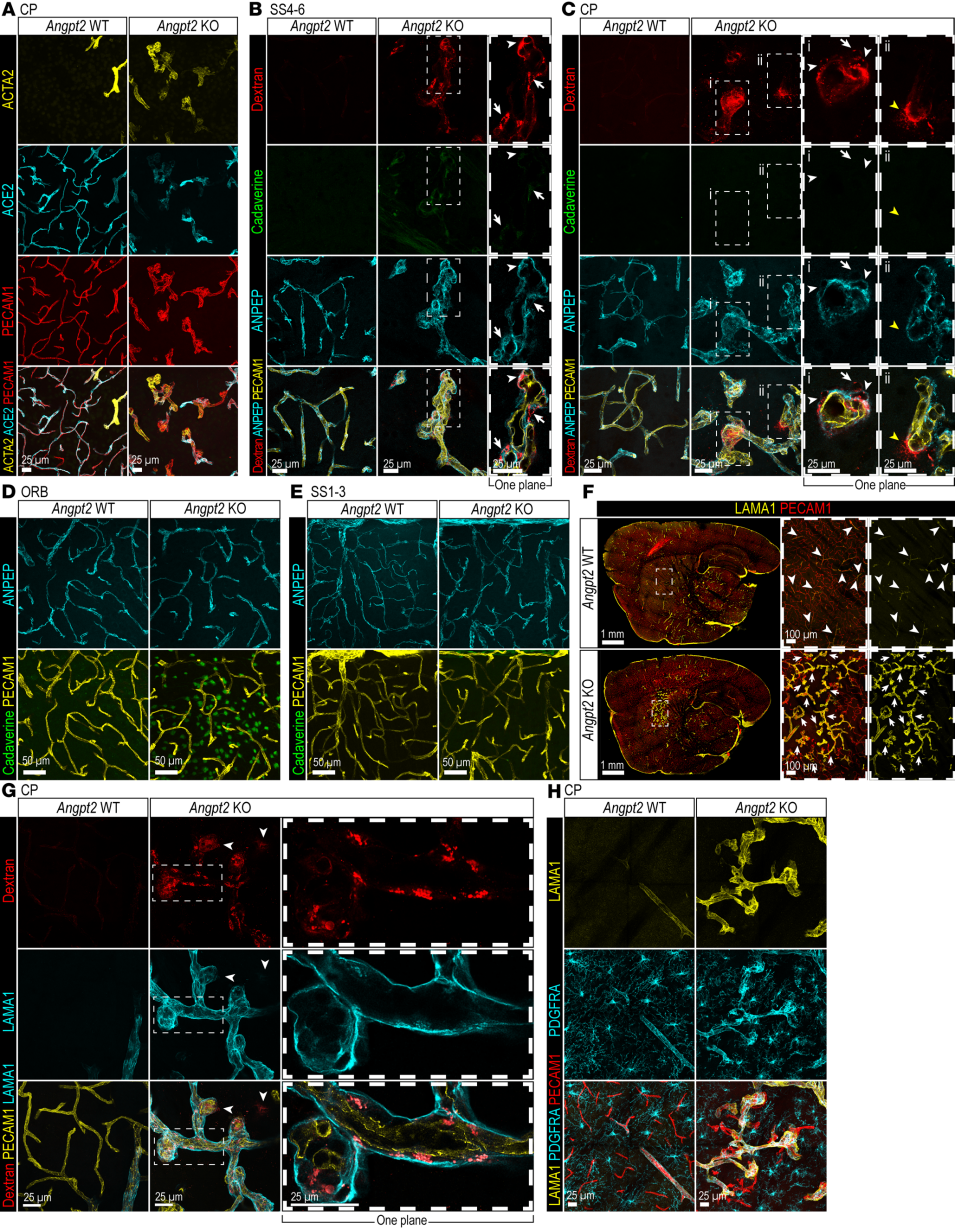
Phenotypes of brain perivascular cells in *Angpt2*-KO brains. (**A**) Representative images of ACE2 (cyan), ACTA2 (yellow), and PECAM1 (red) IF in CP from *Angpt2* WT (*n* = 5) and KO (*n* = 6). Scale bars: 25 μm. (**B** and **C**) Representative images of ANPEP (cyan) and PECAM1 (yellow) IF with 70 kDa TMR-dextran (red) and 1 kDa A488-cadaverine (green) in SS4–6 (**B**) and CP (**C**) (*n* = 4). High-magnification images of 1 *Z*-plane of the tangled vasculature with parenchymal dextran leakage are shown. ANPEP^+^ cells with dextran uptake (white arrowheads); ANPEP^–^ cells with dextran uptake (arrows). Parenchymal 70 kDa TMR-dextran leakage (yellow arrowheads). (**D** and **E**) Representative images of ANPEP (cyan) and PECAM1 (yellow) IF and 1 kDa A488-cadaverine (green) in ORB (**D**) and SS1–3 (**E**) (*n* = 4). Scale bars: 50 μm. (**F**) Representative tile scans of LAMA1 (yellow) and PECAM1 (red) IF on sagittal medial sections of *Angpt2* WT (*n* = 5) and KO (*n* = 6). High-magnification images of malformed vasculature covered by LAMA1^+^ sleeves in KO (arrows) and big vessels covered by LAMA1^+^ sleeves in WT (arrowheads) are shown. Scale bars: 1 mm. (**G**) Representative images of LAMA1 (cyan) and PECAM1 (yellow) IF and 70 kDa TMR-dextran (red) in CP (*n* = 3). Parenchymal TMR-dextran leakage outside LAMA1 sleeve (arrowheads). High-magnification images of 1 *Z*-plane of malformed vasculature covered by LAMA1^+^ sleeves are shown. Scale bars: 25 μm. (**H**) IF of LAMA1 (yellow), PDGFRA (cyan), and PECAM1 (red) in CP from *Angpt2* WT (*n* = 5) and KO (*n* = 6). Scale bars: 25 μm (**B** and **C**); 100 μm (**F**); 25 μm (**G**).

These analyses revealed clearly abnormal mural cell shapes in the regions with malformed vasculature (Figure 3, A and B, and Supplemental Figure 3A). These mural cells displayed abnormally thick processes differing from the pericyte morphology in WT mice (Figure 3A). Some of these abnormal mural cells had taken up extravasated 70 kDa TMR-dextran, yet not all of the dextran^+^ perivascular cells were positive for the mural cell marker aminopeptidase N (ANPEP) (Figure 3, B and C). Some non-malformed KO regions also displayed abnormal pericyte process thickening, although this abnormality was modest in comparison with those observed in the malformed regions (Figure 3, D and E). The occurrence of mural cell abnormalities did not correlate with A488-cadaverine leakage, which was observed in regions both with and without detectable mural cell abnormalities (Figure 3, D and E, and Supplemental Figure 3, D–F).

In the normal mouse brain, expression of the smooth muscle cell marker α-smooth muscle actin (ACTA2) is largely restricted to the mural cells of arteries, arterioles, and large veins, whereas the mural cells of capillaries and venules (i.e., pericytes) are ACTA2^–^ (19). Therefore, we found it intriguing that the mural cells around the malformed vasculature were ACTA2 positive (Figure 3A and Supplemental Figure 3, A and B). This staining pattern was, however, different from that in ACTA2^+^ arterial/arteriolar smooth muscle cells found around arterioles at other locations in both *Angpt2*-WT and -KO brains. The mural cells around malformed vessels also showed downregulated expression of angiotensin-converting enzyme 2 (ACE2), a marker normally expressed in brain pericytes in the mouse. Yet the ACE2 expression levels were normal in the dilated vessels that surround the malformed vasculature (Supplemental Figure 3A). Two additional mural cell markers, platelet-derived growth factor receptor-β (PDGFRB) and desmin (DES), showed increased expression in the regions with malformed vasculature (Supplemental Figure 3, B and C). In summary, mural cell phenotypes were altered in the malformed regions of *Angpt2*-KO brains, whereas those outside the malformed areas showed only modest changes and did not correlate with the presence/absence of 1 kDa A488-cadaverine leakage.

The increased avascular areas in the regions with malformed vasculature of the *Angpt2*-KO brain, along with the elevated expression of ANPEP and PDGFRB — expressed primarily by mural cells but also, to a lesser extent, in perivascular fibroblasts (19) — prompted us to analyze the expression of fibroblast markers. IF for laminin-α1 (LAMA1), a basement membrane component deposited by brain perivascular and pial fibroblasts (19), showed that the majority of the vessels in the regions with malformed vasculature were covered by LAMA1^+^ basement membrane sleeves (Figure 3, F–H), whereas capillaries in the regions with dilated vasculature surrounding the malformations were devoid of LAMA1 sleeves, similar to the situation in WT brains, where LAMA1 was absent from capillaries (Figure 3F). While 70 kDa TMR-dextran deposits were often retained within the LAMA1^+^ basement membrane, it was not uncommon to find the tracer also outside the LAMA1 deposits (Figure 3G). PDGFRA is an established marker for both perivascular fibroblasts and oligodendrocyte progenitor cells (19). IF staining using a PDGFRA antibody demonstrated that fibroblasts expressing this marker were localized around the dilated and tangled vessels within the regions of malformed vasculature besides their expected localization around bigger vessels (Figure 3H). Also, PDGFRA^+^ oligodendrocyte progenitor cells in malformed regions displayed altered morphology in comparison with control brains (Figure 3H); however, these cells were not further characterized.

### Region-specific reactive phenotype of microglia and astrocytes in Angpt2-KO brains.

**Figure 4:**
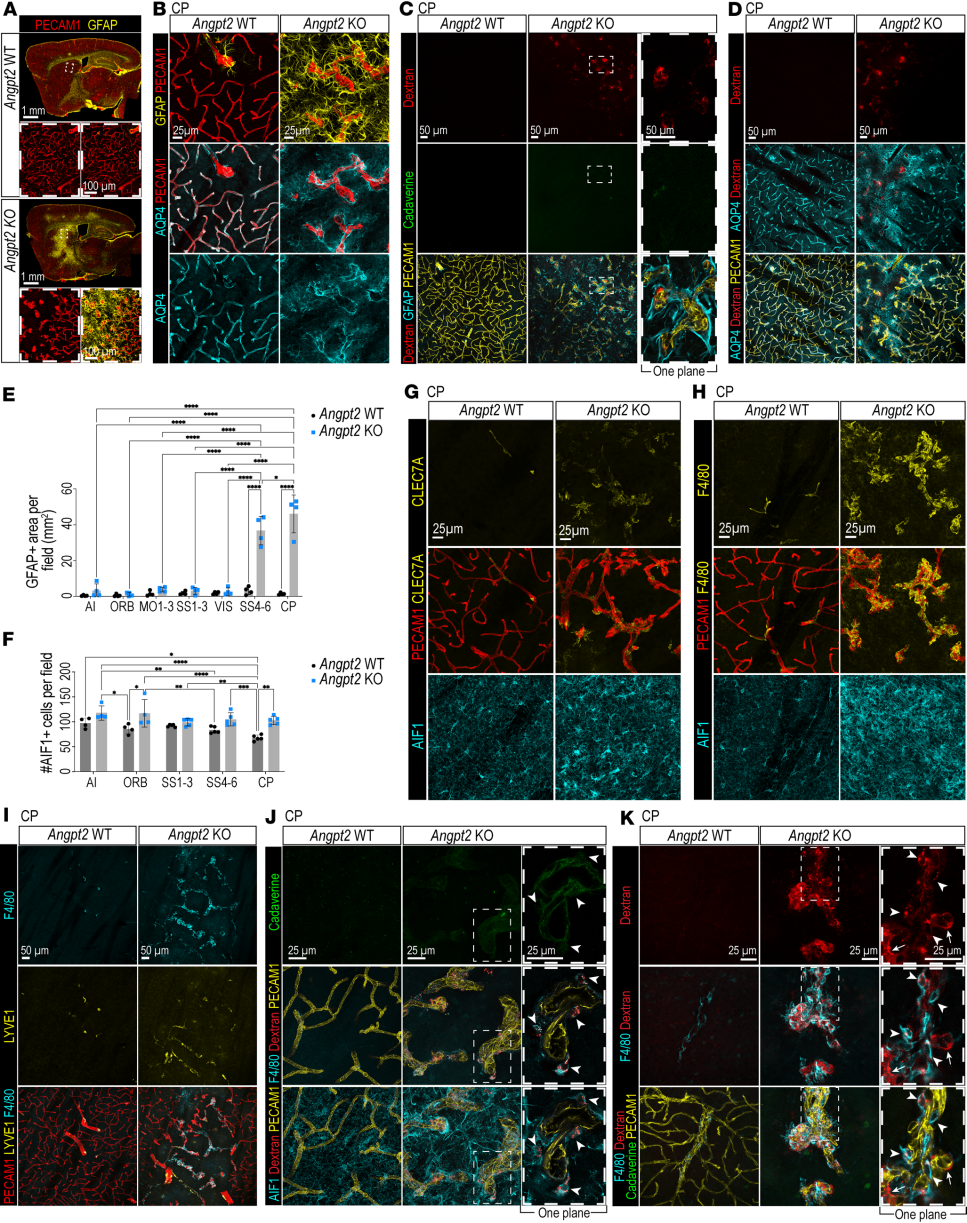
Glial phenotypes in *Angpt2*-KO brains. (**A**) GFAP (yellow) and PECAM1 (red) IF tile scans of Angpt2-WT (*n* = 5) and -KO (*n* = 6) brains. High-magnification: malformed vasculature in CP with reactive GFAP^+^ astrocytes. Scale bars: 1 mm. (**B**) GFAP (yellow), AQP4 (cyan), and PECAM1 (red) IF in CP from WT (*n* = 3) and KO (*n* = 4). Scale bars: 25 μm. (**C**) GFAP (cyan), PECAM1 (yellow), TMR-dextran (red), and A488-cadaverine (green) IF in CP (*n* = 4). High-magnification single *Z*-plane: malformed vasculature and TMR-dextran deposits in between GFAP^+^ astrocytes and ECs. Scale bars: 50 μm. (**D**) AQP4 (cyan), PECAM1 (yellow), and TMR-dextran (red) IF in CP (*n* = 4). Scale: 50 μm. (**E**) GFAP^+^ area quantification in cortical regions and CP (*n* = 4). (**F**) AIF1^+^ cell count per field in cortical regions and CP of WT and KO (AI and ORB, *n* = 4; SS and CP, *n* = 5). Data normally distributed; 2-way ANOVA with Tukey’s test. *P < 0.05, **P < 0.01, ***P < 0.001, ****P < 0.0001. (**G** and **H**) CLEC7A (yellow) (**G**), F4/80 (yellow) (**H**), AIF1 (cyan), and PECAM1 (red) IF in CP from WT (*n* = 3) and KO (*n* = 4). Scale bars: 25 μm. (**I**) LYVE1 (yellow), F4/80 (cyan), and PECAM1 (red) IF in CP from WT (*n* = 3) and KO (*n* = 4). Scale bars: 50 μm. (**J**) AIF1, F4/80 (cyan), PECAM1 (yellow), TMR-dextran (red), and A488-cadaverine (green) IF in CP (*n* = 4) showing TMR-dextran deposits colocalizing with F4/80^+^ and AIF1^+^ cells (arrowheads). Scale bars: 25 μm. (**K**) F4/80 (cyan), PECAM1 (yellow), TMR-dextran (red), and A488-cadaverine (green) IF in CP (*n* = 4). High-magnification single *Z*-plane: tangled vasculature with TMR-dextran deposits colocalizing with F4/80^+^ cells (arrowheads) or without (arrows). Scale bars: 25 μm.

The abnormalities in ECs, mural cells, and perivascular fibroblasts in *Angpt2*-KO brains encouraged us to also study the glial cells. Region-specific massive upregulation of glial acidic fibrillary protein (GFAP), a marker of reactive astrocytes, was observed in KO brains (Figure 4A and Supplemental Figure 4A). GFAP expression was prominent specifically in regions with vascular malformations and adjacent regions with dilated vasculature (Figure 4, A–C, and Supplemental Figure 4, A and B). To get a better overview of the GFAP reactive phenotype in the *Angpt2*-KO brains and to find out whether it matched with the different types of tracer leakage, we quantified the GFAP area per field in AI, ORB, MO layers 1–3, SS1–3, SS4–6, and VIS and CP regions. Only the KO brain regions with malformed vasculature and 70 kDa TMR-dextran leakage (SS4–6 and CP) (Figure 2C) displayed elevated GFAP expression, whereas non-malformed regions, regardless of A488-cadaverine leakage, had normal (mostly low) GFAP expression (Figure 4E). Another astrocyte marker, the water channel aquaporin-4 (AQP4), is normally concentrated at the astrocyte end-foot membrane facing the vasculature. However, in *Angpt2* KO, AQP4 was partially mislocalized to other subcellular regions of the astrocytes around the malformed vasculature with 70 kDa TMR-dextran leakage (Figure 4, B and D, and Supplemental Figure 4, D and E), whereas the normal end-foot–restricted localization was observed in non-malformed regions in the KO comparably to WT (Supplemental Figure 4, D and E). Thus, both GFAP and AQP4 stainings suggested that astrocyte reactivity correlates with vascular malformations and 70 kDa TMR-dextran leakage.

We next assessed microglial abundance and morphology by staining for allograft inflammatory factor 1 (AIF1). Analysis of AI, ORB, SS1–3, SS4–6, and CP regions revealed increased numbers of AIF1^+^ cells in ORB and CP regions (Figure 2, C and D, and Figure 4F), while the differences in AIF1^+^ cell numbers in other regions were not statistically significant when *Angpt2* WT was compared with KO. In regions with malformed vasculature in *Angpt2* KO, AIF1^+^ cells were unevenly distributed and displayed a reactive phenotype including thickening, shortening, and asymmetric distribution of cellular processes in comparison with *Angpt2* WT (Figure 4, G, H, and J, and Supplemental Figure 4F). In KO regions with macroscopically normal vasculature and increased A488-cadaverine leakage, the AIF1^+^ cell morphology was comparable to that in WT controls (Supplemental Figure 4G). Staining for the macrophage markers C-type lectin domain family 7 member A (CLEC7A) and F4/80 in combination with AIF1 showed that these were present in cells tightly surrounding the malformed vasculature in *Angpt2*-KO brains (Figure 4, G and H, and Supplemental Figure 4F). As expected, CLEC7A and F4/80 were also expressed by perivascular macrophages (20, 21). This was observed both in *Angpt2*-WT brains and in non-malformed regions with dilated vasculature in KO brains (Figure 4, G–K, and Supplemental Figure 4, G–I). Upregulation of these proteins in reactive microglia has been reported previously (22, 23). However, because of the proximity of these cells to the vasculature, we cannot definitively distinguish perivascular macrophages from reactive microglia surrounding the malformed vessels. Costainings of F4/80 and the resident perivascular macrophage marker lymphatic vessel endothelial hyaluronan receptor-1 (LYVE1) (21) confirmed the presence of LYVE1^+^ perivascular macrophages in malformed regions enriched with bigger vessels (Figure 4I). However, many F4/80^+^ cells around the malformed vessels were LYVE1^–^. Numerous F4/80- and AIF1^+^ perivascular cells surrounding the vascular malformations were positive for the 70 kDa TMR-dextran tracer, suggesting they had internalized it (Figure 4, J and K). This suggests that both pericytes and microglia/macrophages can phagocytose extravasated 70 kDa TMR-dextran — unlike the extravasated 1 kDa A488-cadaverine, which was instead taken up by neurons and possibly also some non-neuronal parenchymal cell, yet to be identified.

### ScRNA-seq reveals a distinctive angiogenic gene expression profile in Angpt2-KO ECs.

**Figure 5:**
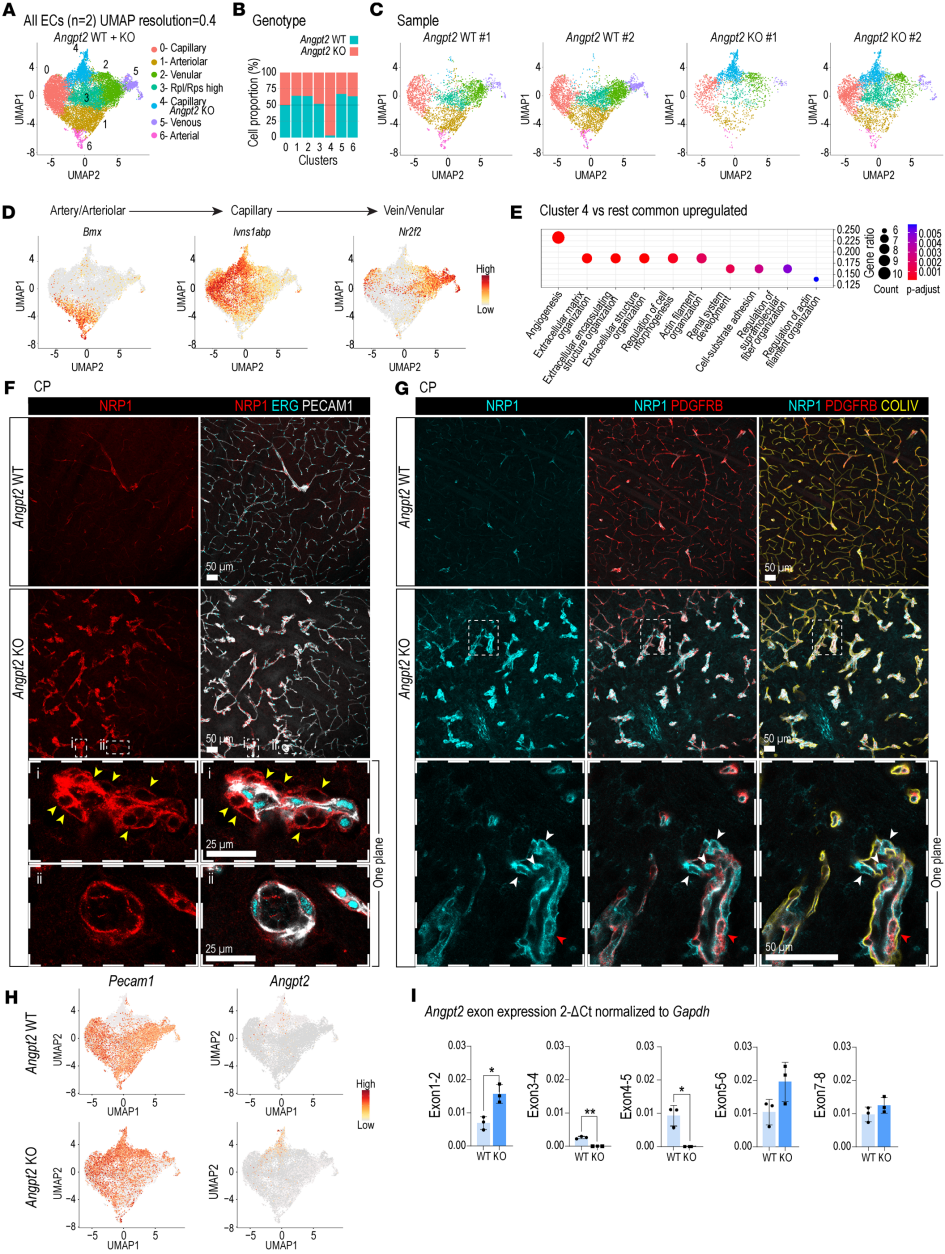
ScRNA-seq analysis in *Angpt2*-WT and -KO mice. (**A**) UMAP of EC dataset, clustered into 7 clusters (0 to 6) at a resolution of 0.4. (**B**) Proportion of ECs in each cluster (0 to 6). (**C**) UMAPs of the cell clusters from each sample. (**D**) UMAPs showing the expression of arteriovenous markers. (**E**) Top 10 GO biological process terms overrepresented in cluster 4. Count = number of genes per term. (**F**) Representative images of NRP1 (red), ERG (cyan), and PECAM1 (gray) IF in CP (*n* = 3). High-magnification images of 1 *Z*-plane magnification of tangled vasculature with EC and perivascular NRP1 expression (yellow arrowheads [i] and NRP1^+^ dilated vasculature [ii]). Scale bars: 25 μm (**F**); 50 μm (**G**). (**G**) Representative images of NRP1 (cyan), PDGFRB (red), and COLIV (yellow) IF in CP (*n* = 3). High-magnification images of 1 *Z*-plane magnification of the dilated vasculature. NRP1^+^/PDGFRB^+^ cells (red arrowheads); NRP1^+^ cells outside the collagen sleeve (white arrowheads). Scale bars: 50 μm. (**H**) UMAPs of *Angpt2* expression. (**I**) Relative mRNA expression of *Angpt2* exons in adult brain microvascular fragments from WT (light blue) and KO (dark blue) mice (*n* = 3). Shown values are 2^–ΔCt^ of *Gapdh*. Data were normally distributed, and a Welch’s *t* test was performed to evaluate significance. *P < 0.05, **P < 0.01.

We hypothesized that the regional EC abnormalities (malformations and dilations) observed in *Angpt2* KO might be accompanied by changes in gene/protein expression. To address this in an unbiased fashion, we performed scRNA-seq analysis. We isolated vascular cells from cerebra of 3-month-old *Angpt2*-WT and -KO mice using a vascular fragment immune-panning protocol (24) and performed scRNA-seq analysis using the 10x Genomics platform. In total, 18,002 single-cell transcriptomes passing quality thresholds were obtained, most of which originated from ECs, but small clusters of other cell types were also identified as expected (microglia, mural cells, and different types of immune cells) (Supplemental Figure 5, A–L, and Supplemental Table 2). EC single-cell transcriptomes were distinguished from those of other cells using canonical EC markers (e.g., *Pecam1*, *Cdh5*, *Kdr*, *Tek*, and others) and subclustered, resulting in 7 endothelial subclusters comprising, in total, 14,120 EC single-cell transcriptomes (Figure 5A and Supplemental Table 3). Notably, EC cluster 4, which displayed a distinct gene expression pattern including many unique genes, consisted of 96% *Angpt2*-KO cells (Figure 5, A–C). Cells from 2 independent experiments distributed similarly across most clusters, indicating minimal batch effects (Figure 5C). The cells were predominantly organized in an arteriovenous zonation pattern, as judged by the distribution of markers for arterial, capillary, and venous brain ECs, as previously described (19) (Figure 5D). The *Angpt2* KO–dominated cluster 4 displayed capillary identity. A searchable database with gene expression patterns for all genes across clusters in *Angpt2*-WT and -KO ECs is available, providing gene-by-gene bar plot visualization (https://andaloussimaelab.org/publications/ang2/database.html).

To provide initial insight into the type of genes and gene functions associated with *Angpt2* KO–dominated cluster 4, we performed differentially expressed gene (DEG) analysis between cluster 4 and the other endothelial clusters (0–3, 5, and 6) (Supplemental Table 4). Gene Ontology (GO) analysis (using the biological process sub-ontology) of upregulated DEGs in cluster 4 revealed enrichment of terms associated mainly with angiogenesis and ECM organization (Figure 5E and Supplemental Table 5). One of the upregulated DEGs specifying the GO term “angiogenesis” was *Nrp1* encoding neuropilin-1 (NRP1), which was upregulated also at the protein level. NRP1 IF was low in WT capillaries (in comparison with arterioles and venules) but elevated in *Angpt2*-KO regions with malformed vasculature (Figure 5, F and G). The NRP1 IF was also higher in perivascular cells surrounding the malformations and located outside the collagen type IV (COLIV) basement membrane sleeve (Figure 5G), which could be attributed to expression in abnormal mural cells (Figure 3), fibroblasts (Figure 3), or microglia/perivascular macrophages (Figure 4) that envelop the malformed vessels in *Angpt2*-KO mice.

*Angpt2* was among the significantly upregulated genes in cluster 4, which may appear paradoxical since the gene is inactivated (Figure 5H and Supplemental Table 4). However, the *Angpt2*-KO mice used in this study carry a selective deletion of exon 4, which encodes a critical part of the ANGPT2 protein (25), whereas 10x Genomics sequencing chemistry is designed to capture the 3′ end of the mRNA through poly(A) priming, and would therefore be expected to detect *Angpt2* mRNA molecules lacking exon 4. To test this assumption, we conducted qPCR analysis for all eight *Angpt2* exons in isolated brain microvascular fragments from *Angpt2*-WT and -KO mice (Figure 5I). These analyses confirmed deletion of exon 4 in *Angpt2*-KO brain but also revealed a modest upregulation of the mutant *Angpt2* transcript, which is not surprising considering the angiogenic phenotype in *Angpt2*-KO mice (Figure 5I).

### Endothelial angiogenic heterogeneity in Angpt2-KO malformed vessels.

**Figure 6:**
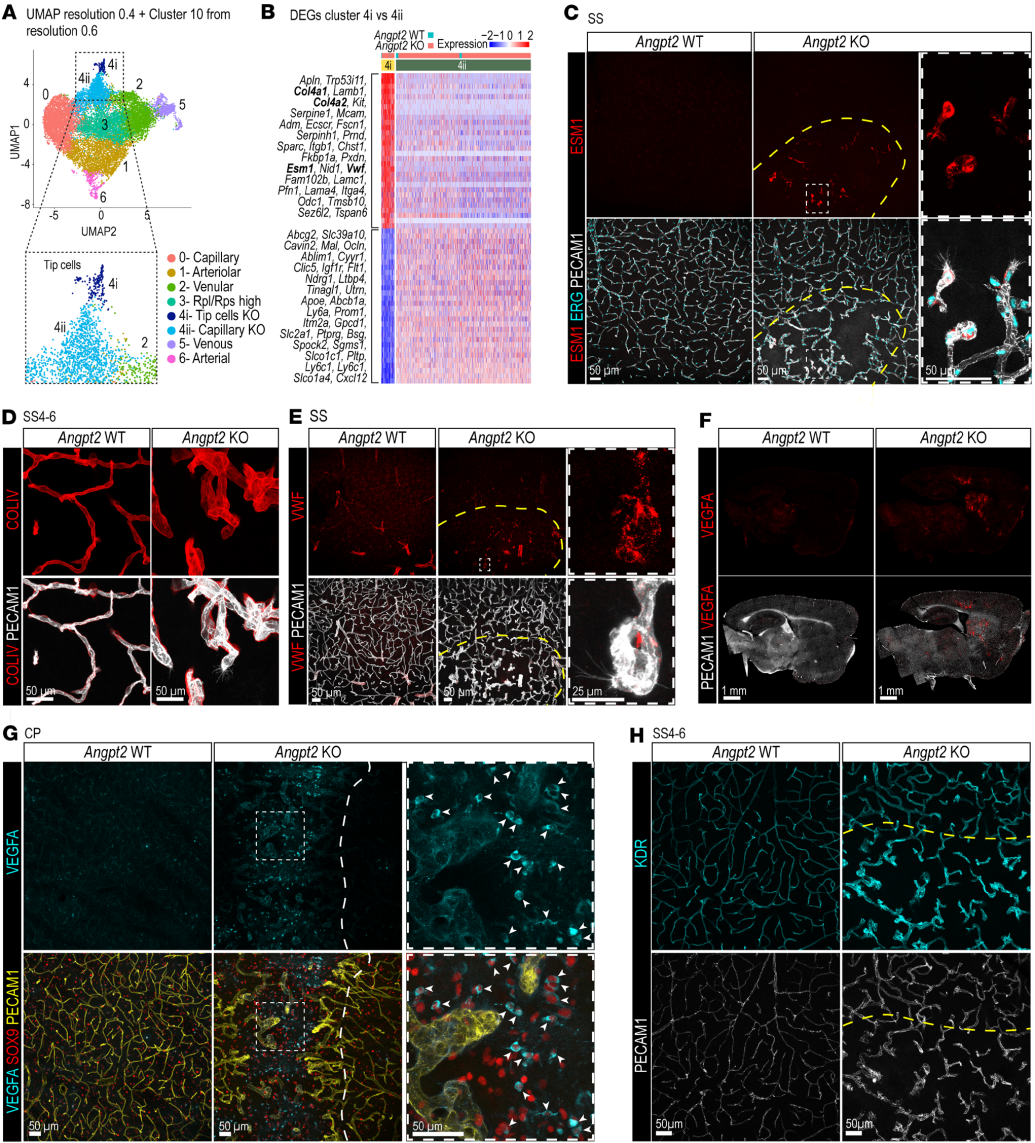
Characterization of angiogenic transcriptional changes in *Angpt2*-WT and -KO brains. (**A**) UMAP of EC dataset, clustered into 8 clusters after further cell separation of cluster 4 (4i and 4ii) using resolution = 0.6 (see Supplemental Figure 6A). (**B**) Top 30 genes from DEG comparison between cluster 4i and 4ii (see Supplemental Table 6). (**C**) Representative images of ESM1 (red), ERG (cyan), and PECAM1 (gray) IF in SS (*n* = 3). High-magnification images of filopodia^+^ and -negative tip cells expressing ESM1. Scale bars: 50 μm (**C** and **G**); 25 µm (**E**). (**D**) Representative images of COLIV (red) and PECAM1 (gray) IF in SS4–6 (*n* = 3). High-magnification images of malformed vasculature with tip cells. Scale bars: 50 μm. (**E**) Representative images of VWF (red) and PECAM1 (gray) IF in SS4–6 (*n* = 3). High-magnification images of VWF expression in tangled malformed vasculature with filopodia. Scale bars: 50 μm. (**F**) Representative images of VEGFA (red) and PECAM1 (gray) IF in medial sagittal sections (*n* = 5). Scale bars: 1 mm. (**G**) Representative images of VEGFA (cyan), PECAM1 (yellow), and SOX9 (red) IF in CP (*n* = 5). High-magnification images of a region with malformed vasculature; SOX9/VEGFA^+^ astrocytes (arrowheads). Scale bars: 50 μm. (**H**) Representative images of KDR (cyan) and PECAM1 (gray) IF in SS4–6 of *Angpt2*-WT and -KO brains (*n* = 3). Scale bars: 50 μm. Dashed lines in **C**, **E**, **G**, and **H** mark rough boundaries between malformed and non-malformed vasculature.

To explore heterogeneity within *Angpt2*-KO cluster 4, we reclustered the dataset at a higher resolution (0.6), resulting in 12 clusters (Supplemental Figure 6A). This identified a subcluster 4i within cluster 4 (Figure 6A and Supplemental Table 6). DEG analysis between 4i and 4ii (Figure 6B and Supplemental Table 7) revealed that cluster 4i exhibited a tip cell profile (including upregulated expression of, e.g., *Esm1*, *Apln*, *Mcam*, *Lamb1*) (11), presumably corresponding to the abundant tip cells observed in the malformed vascular regions (Figure 1, C and H). Indeed, IF stainings with ESM1 antibody labeled abundant tip cells, both with and without filopodia, in the malformed vessels (Figure 6C and Supplemental Figure 6B). In addition to previously reported tip cell transcriptomic profiles (11, 26, 27), we identified several additional tip cell markers, including *Col4a1* and *Col4a2* and *Vwf* (Figure 6B). IF stainings using COLIV and VWF antibodies confirmed these transcriptional changes at the protein level (Figure 6, D and E). Biological process GO analysis of DEGs in both subclusters confirmed that 4i represents sprouting tip cells, and that the entire cluster 4 maintains an overall angiogenic transcriptional signature (Supplemental Figure 6C and Supplemental Table 8). Assessment of a range of cell cycle markers (28) confirmed our MKI67 IF analysis (Figure 1, I and J), showing that all tip cells and most of the ECs in *Angpt2*-KO mice were non-proliferative (Supplemental Figure 6D).

Increased angiogenesis in the malformed vasculature prompted us to analyze whether this was accompanied by vascular endothelial growth factor A (VEGFA) expression. Indeed, IF stainings revealed that strong VEGFA signal colocalized with the regions of vascular malformations in the *Angpt2*-KO brains (Figure 6, F and G, and Supplemental Figure 6E). It was upregulated in the CP and SS4–6 regions, where the biggest malformed regions are found. Additionally, the distinct VEGFA expression pattern also revealed small malformations throughout the brain. A subpopulation of SOX9^+^ astrocytes and some NEUN^+^ neurons colocalized with VEGFA expression (Figure 6G and Supplemental Figure 6E). Furthermore, VEGFA immunoreactivity delineated the ECs within the malformed vasculature of *Angpt2*-KO brains. Because scRNA-seq did not show any upregulation of *Vegfa* in ECs, the detected protein was most likely derived from astrocytes and neurons (Figure 6G and Supplemental Figure 6E). Not surprisingly, the main receptor for VEGFA, KDR (VEGFR2), was also upregulated in the malformed vasculature (Figure 6H), as was the coreceptor NRP1 (Figure 5, F and G).

### Tie signaling pathway changes in response to vascular malformations.

**Figure 7:**
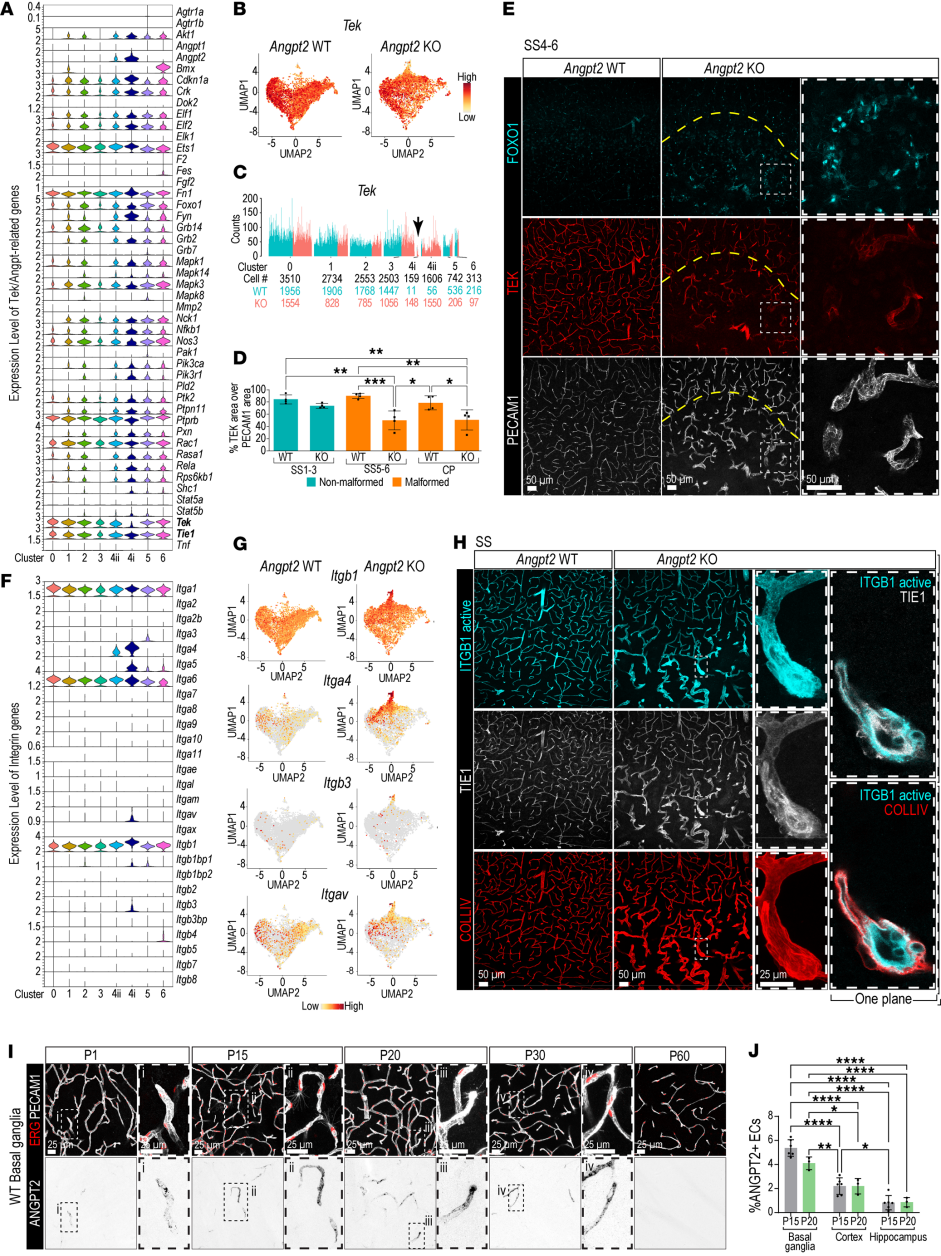
Characterization of Angpt/Tek and integrin changes in *Angpt2*-WT and -KO brains. (**A**) Violin plot of Tek/Angpt2-related gene expression in different EC clusters. (**B**) UMAPs of *Tek* expression. (**C**) Bar plots illustrating *Tek* expression. Cluster 4i with low *Tek* expression (arrow). (**D**) Percentage TEK/PECAM1 area in non-malformed and malformed regions (*n* = 4). Data were normally distributed, and a 1-way ANOVA with Tukey’s multiple-comparison test was performed to evaluate significance. *P < 0.05, **P < 0.01, ***P < 0.001. (**E**) Representative images of TEK (red), FOXO1 (cyan), and PECAM1 (gray) IF in SS4–6 (*n* = 3). High-magnification images of regions with malformed vasculature, low TEK expression, and nuclear FOXO1 expression. Dashed line marks rough boundary between malformed and non-malformed vasculature. Scale bars: 50 μm (**E**); 25 μm (**H** and **I**). (**F**) Integrin-encoding genes in different EC clusters. (**G**) UMAPs illustrating *Itgb1*, *Itga4*, *Itgb3*, and *Itgav* expression. (**H**) Representative images of active-ITGB1 (cyan), TIE1 (gray), and COLIV (red) IF in SS (*n* = 3). High-magnification images of malformed vessel with high active-ITGB1 expression in perivascular and endothelial cells. One *Z*-plane overlay of active ITGB1 with TIE1 and COLIV. Scale bars: 50 μm. (**I**) Spatiotemporal ANGPT2 expression in C57BL/6J at P1–60. ERG (red), PECAM1 (gray), ANGPT2 (black). High-magnification images of the ANGPT2^+^ vasculature (*n* = 3). Scale bars: 25 μm. (**J**) Quantification of ANGPT2^+^ ECs in P15 (gray) and P20 (green) C57BL/6J in basal ganglia, cortex, and hippocampus (*n* = 3). Data were normally distributed, and a 2-way ANOVA with Šidák’s multiple-comparison test was performed to evaluate significance. *P < 0.05, **P < 0.01, ****P < 0.0001.

ANGPT2 has been shown to signal either through the TEK/TIE2 receptor (5, 9, 10, 29) or via integrins (8, 30), in a context-dependent manner. To investigate how *Angpt2* deletion affects the two ANGPT signaling pathways, we first analyzed the expression patterns of genes involved in the ANGPT/TEK signaling pathway (https://www.gsea-msigdb.org/gsea/msigdb/cards/PID_ANGIOPOIETIN_RECEPTOR_PATHWAY). Notably, *Tek* transcript levels were reduced in the *Angpt2* KO–dominated tip cell cluster 4i (Figure 7, A–C), in agreement with previous observations in postnatal retinal angiogenesis (8), while *Tie1* expression appeared unchanged (Figure 7A). IF staining confirmed these transcriptomic findings, with TEK antibody staining validating the observed TEK reduction in the tip cells in the regions with malformed vasculature (Figure 7, D and E). Interestingly, TIE1 immunostaining appeared higher in the malformed vessels. However, analysis at higher magnification showed non-vascular TIE1 staining in cells surrounding the malformations, which might represent ACTA2^hi^ACE2^lo^ abnormal mural cells since these cells reside within the COLIV sleeve (Figure 7H). Forkhead box O1 (FOXO1), a transcription factor known to drive *Angpt2* expression (31, 32), was slightly lower in cluster 4i (Figure 7A; see also the online database), and immunostainings revealed strong nuclear staining in the regions with malformed vasculature (Figure 7E). Translocation of FOXO1 to the nucleus is indicative of decreased TEK phosphorylation (31), which is also in agreement with the upregulated *Angpt2* mRNA expression in *Angpt2*-KO brains (Figure 5, H and I). In retinal angiogenesis, *Angpt2* signaling has been reported to occur via integrins (8). We therefore examined the expression of all identified integrins (Itg) across all clusters. While *Itga4* and *Itgb1* displayed differential expression in the *Angpt2* KO–dominated EC cluster 4 compared with WT cells, *Itgb3* and *Itgav* showed specific upregulation in the tip cell–specific subcluster 4i (Figure 7, F and G). IF stainings with ITGB1 antibody confirmed the transcriptional findings (Figure 7H).

We have previously shown that expression of ANGPT2 in the adult brain vasculature is virtually nonexistent (11–13); therefore we investigated its spatiotemporal expression in the WT postnatal brains. These analyses revealed a transient expression window, with ANGPT2 peaking at P15–20 and becoming largely undetectable by P60 (Figure 7, I and J, and Supplemental Figure 7, A and B). It was low in hippocampus and cortex and highest in basal ganglia. These findings indicate that the angiogenic phenotype and related vascular abnormalities are probably established during postnatal brain development.

### Transcriptional changes in different BBB leakage models.

Previously, we demonstrated that the pericyte-deficient *Pdgfb^ret/ret^* mouse model, which exhibits BBB permeability to tracers of 1–200 kDa (11, 18, 33), also shows increased vascular sprouting without concurrent endothelial proliferation (11). ScRNA-seq analysis of ECs from *Pdgfb^ret/ret^* mouse brains revealed that ECs lacking pericyte contact retain a general BBB-specific gene expression profile but acquire a venous-shifted molecular pattern and exhibit extensive changes in growth factor and regulatory protein expression (11). To obtain initial insight into potential similarities between *Angpt2*-KO malformed ECs and *Pdgfb^ret/ret^* transformed ECs, we extracted the marker genes from *Pdgfb^ret/ret^* transformed ECs (11) and studied their expression in *Angpt2*-KO cluster 4 malformed cells. While both mouse mutants shared a similar, albeit not identical, tip cell profile (Supplemental Figure 8A and Supplemental Table 9), including the upregulated expression of several growth factors and growth factor–regulatory proteins (e.g., *Fgfbp1*, *Ccn2*, *Bmp6*), *Angpt2*-KO ECs maintained a capillary signature without the venous shift observed in *Pdgfb^ret/ret^* vessels. We next examined the expression of 64 BBB markers regulated by Wnt signaling (34, 35) in *Angpt2*-KO cluster 4 and found that all except four remained expressed at normal levels; two were upregulated (*Vwa1*, *Nrp1*) and two were downregulated (*Slc38a5*, *Slc16a1*) (Supplemental Figure 8B). Also, this result was similar between *Pdgfb^ret/ret^* transformed ECs and *Angpt2*-KO cluster 4 malformed cells, suggesting that vascular malformation and angiogenic sprouting can occur on top of a largely intact BBB gene expression profile.

## Discussion

Elevated ANGPT2 levels have been observed in various mouse models of brain vascular defects (11–14) as well as in human glioblastoma (15, 16) and stroke (17). Also, constitutive *Angpt2* loss leads to vascular abnormalities and BBB leakage (11). In this study, we extended these analyses to gain deeper insight into the nature of vascular malformations and BBB leakage in *Angpt2*-KO mice.

Our findings revealed that *Angpt2* KO leads to vascular malformations primarily in 2 regions: the SS layers 4–6 and the CP. These malformed vessels were dilated and surrounded by avascular areas of increased size. Further detailed analysis of cleared brain sections showed that while some vessels were abnormally dilated and bulged, the majority formed slightly dilated, intertwined structures resembling loose, ball-like vessel networks. The intertwined nature of the tangled vessels made it challenging to measure and quantify the vascular length in the malformed vasculature of *Angpt2*-KO brain, and we therefore quantified EC numbers using the nuclear EC-specific marker ERG. Despite the presence of increased avascular areas in *Angpt2*-KO regions with malformed vasculature, the EC numbers did not differ in these regions between the *Angpt2*-WT and -KO mice. The tangled vessels were characterized by abundant tip cells laden with protruding filopodia, yet this was not accompanied by the formation of regular angiogenic sprouts, or by EC proliferation; the latter was also confirmed by scRNA-seq. We have previously observed vascular sprouting without increased EC proliferation in adult pericyte-deficient mice (11). The tip cell signature partly overlapped with previously proposed tip cell markers (11, 26, 27); however, there were also several notable differences, such as expression of Col4a1, Col4a2, and Vwf. Whether these differences are real or are caused by method bias requires further analyses. Importantly, the malformed vasculature was surrounded by distinct subsets of VEGFA^+^ astrocytes and neurons, and its receptor VEGFR2 (KDR) (36, 37) and coreceptor NRP1 (38) were upregulated in the malformed ECs when compared with the non-malformed vasculature. NRP1 was also upregulated in perivascular cells surrounding the malformations. Whether disturbances of ANGPT2/VEGFA/VEGFR2/NRP1 signaling are the leading cause of vascular malformations and BBB permeability in *Angpt2*-KO brains remains to be determined.

We show that *Angpt2*-KO brain regions with malformed vasculature exhibit permeability to exogenous tracers ranging from 1 to 70 kDa, as well as to erythrocytes. Additionally, we consistently detected 1 kDa A488-cadaverine extravasation in areas with macroscopically normal-appearing vessels. In our previous work, we reported reduced CLDN5 expression in *Angpt2*-KO brain vessels (11). However, in the current study, using improved IF protocols, we observed that CLDN5 distributed along EC junctions comparably to CDH5 distribution despite the irregular junctional morphology in malformed vessels.

Previously, we reported that pericyte-deficient mice exhibited BBB leakage of tracers ranging from 1 to 200 kDa (11, 13, 18). Therefore, it was unexpected that the malformed vessels in *Angpt2*-KO mice, which showed leakage of both 1 kDa A488-cadaverine and 70 kDa TMR-dextran, were characterized by abnormally high mural cell coverage. Notably, the normal-appearing vasculature within regions showing only A488-cadaverine leakage also lacked signs of pericyte loss, although subtle but consistent changes in pericyte morphology were observed. Our vascular cell isolation protocol for scRNA-seq was designed to enrich primarily ECs, recovering only limited numbers of mural cells. Thus, the analysis of mural cell heterogeneity by scRNA-seq was limited by low cell numbers. Nevertheless, the observation that both pericyte deficiency and excessive mural cell coverage can lead to BBB leakage suggests the existence of an optimal threshold for pericyte coverage necessary to maintain BBB integrity.

The tangled vessels in the malformation regions were not only covered by mural cells but also completely ensheathed by fibroblasts, thick ECM sleeves rich in COLIV and LAMA1, reactive microglia/macrophages, and AQP4^–^ and GFAP^+^ astrocyte end-feet. Despite the observed vascular leakage, we observed no signs of seizures or increased lethality in these mice, in contrast to loss of *Cldn5*-dependent leakage (12). It is possible that these additional cellular layers and the increased ECM serve a protective role for surrounding neurons. Alternatively, the leakage may occur primarily through transcytosis — a mechanism previously reported to be tolerated and non-lethal in experimental mice (11, 18) — whereas leakage through disrupted tight junctions has been associated with lethality (12). Another noteworthy finding was the partial mislocalization of AQP4 away from astrocyte end-feet, which may suggest disrupted water transport in these end-feet; however, this phenomenon was not explored further in the current study. While GFAP^+^ reactive astrocytes were predominantly localized to areas with malformed vasculature showing 70 kDa TMR-dextran leakage, they also extended into regions with dilated but otherwise normal-appearing vessels. In contrast, GFAP expression was not elevated in areas with leakage of only 1 kDa A488-cadaverine. In summary, these findings suggest a complex glial and ECM response to vascular abnormalities, which warrants further investigation.

TEK, the primary ANGPT2 receptor, displayed a weaker expression pattern in regions with malformed vasculature, with reduced or absent levels in sprouting ECs. Also, the nuclear translocation of FOXO1 in ECs suggests that TEK phosphorylation is decreased (31). In contrast, TIE1 expression remained unchanged in the endothelium; however, we could detect its expression in non-endothelial cells in the *Angpt2*-KO malformed regions. Several integrins were upregulated in tip cells of the malformed vessels, though their functional significance remains unclear. Strikingly, *Angpt2*-KO brain regions with malformed vasculature exhibited increased mural cell coverage, underscoring the importance of ANGPT2 in endothelial-pericyte crosstalk. While pericyte-derived ANGPT1 signaling via TEK is known to regulate vascular remodeling, maturation, and stabilization (3, 4), the role of endothelial cell–derived ANGPT2 in pericyte signaling remains unknown. It is important to note that we were analyzing 2- to 3-month-old constitutive-KO mice and that ANGPT2 is normally low or missing in the brain at this age. While transcriptomic analysis provided insights into the malformed vasculature phenotype, it does not reveal the cell-autonomous function of ANGPT2 in brain ECs. We assume that further mechanistic insights in ANGPT2-regulated signaling pathway(s) should be revealed through detailed follow-up studies at the relevant postnatal ages using constitutive- and conditional-KO mice. Furthermore, additional research is needed to determine whether the observed increase in perivascular cell coverage and astrocyte/microglia reactivity results from BBB leakage, as seen in *Cldn5^iECKO^* mice (12), or whether other molecular or cellular mechanisms are involved.

In conclusion, we have uncovered an unexpected cerebrovascular function of ANGPT2, the absence of which not only affects the vascular endothelium but has profound consequences also for the organization of mural cells, perivascular fibroblasts, astrocytes, and microglia. Our results further uncover distinct modes of BBB leakage and tracer handling by brain parenchymal cells.

## Methods

### Sex as a biological variable.

Our study examined male and female animals, and similar findings were obtained for both sexes.

### Experimental mice.

The *Angpt2* constitutive KO allele was generated by crossing of *Angpt2^flox^* mice (25) with a *Pgk*-Cre mouse line (39). The heterozygote *Angpt2* mice were backcrossed at least 10 generations with C57BL6/J (The Jackson Laboratory) mice. The experimental mice were created through heterozygous breedings, and their WT littermates were used as controls at 2–3 months of age. C57BL6/J mice were used for ANGPT2 expression analyses. Experiments have been reported in compliance with the ARRIVE 2.0 guidelines (40). All efforts were made to minimize animal suffering. The blinded analyses were impossible due to striking vascular phenotypes in *Angpt2* KO.

### IF stainings.

Mice were anesthetized with 20 mg/mL ketamine and 2 mg/mL xylazine in 1× phosphate-buffered saline (PBS), followed by transcardial perfusion with Hanks balanced salt solution (HBSS) and 4% formalin prior to brain collections. The brains were postfixed in 4% formalin for 4 hours at 4°C and cut into 75 μm sagittal sections with vibratome (Microm HM650V, Thermo Fisher Scientific). The mice used for CLDN5, ZO1, ACE2, ACTA2, NRP1, KDR, TIE1, TEK, and ITGB1 immunostainings were perfused with HBSS followed by 1% formalin, and the brains were collected and incubated in ice-cold 100% methanol for 2–4 hours at 4°C and sectioned after gradual rehydration (75%, 50%, 25%, 0% methanol in PBS, each step 20 minutes at 4°C). The sections were incubated in permeabilization and blocking buffer (PBS with 1% bovine serum albumin [BSA], 5% normal donkey serum [catalog 017-000-121, Jackson ImmunoResearch], 0.75% Triton X-100) overnight at 4°C. Primary antibodies (diluted in 0.5% BSA, 0.25% Triton X-100 in PBS) were incubated 48–72 hours at 4°C, followed by an overnight secondary antibody incubation at 4°C. The antibodies used are listed in Supplemental Table 1. The stained sections were mounted in ProLong Gold antifade reagent (catalog P36930, Thermo Fisher Scientific). Images were taken with a Leica TCS SP8 confocal microscope (Leica Microsystems), and are shown in the figures as maximum-intensity projections of *Z*-stacks or as single planes. Detailed images (Figure 3, B and G, and Figure 4J) were taken with a ×63 objective with the recommended settings according to the Nyquist calculator (https://svi.nl/Nyquist-Calculator). The images were then deconvoluted using Huygens Essential software with the express deconvolution tool. Image analysis was done using Fiji (ImageJ, NIH), image brightness levels and sizing were adjusted using Adobe Photoshop v25.11, and figures were built using Adobe Illustrator v27.5.

### Tissue clearing.

After IF staining, 250-μm-thick sections were optically cleared using the F-CUBIC protocol (41). The sections were incubated at room temperature in F-CUBIC solution [10 wt% formamide, 25 wt% urea, 25 wt% *N*,*N*,*N*′,*N*′-tetrakis (2-hydroxypropyl)ethylenediamine, and 15 wt% Triton X-100 in water] until sections became transparent (10–30 minutes). All image stacks were taken with a Leica TCS SP8 confocal microscope (Leica Microsystems). Sequential 2-dimensional image stacks were 3D-rendered using Imaris (Bitplane) and vessel volume outlines generated with the surface tool. 3D volumes are depicted in Supplemental Video 1 and the original 2D image stacks in Supplemental Video 2. Maximum projection of the volume outlines is shown in Figure 1.

### Quantifications.

A minimum of 4 fields from 2 sections per mouse were analyzed from malformed regions (SS4–6, CP) and non-malformed regions (SS1–3, MO) with Fiji.

For avascular space and GFAP^+^ and TEK^+^PECAM1^+^ areas, maximum-intensity projections of *Z*-stacks were thresholded, using the same threshold value for both *Angpt2*-WT and -KO images. Subsequently, binary image areas (μm^2^) per field were measured. For avascular area, PECAM1^+^ capillary area was subtracted from the total field area.

ERG^+^ cells, tip cells with filopodia, and MKI67^+^, MKI67^+^ERG^+^, and AIF1^+^ cells were counted manually from maximum-intensity projections of *Z*-stacks.

The mean distance between vessels per field was calculated from PECAM1 binary images used to create distance maps in Fiji.

### BBB permeability assessment.

Seventy-kilodalton TMR-dextran (catalog D1818, Thermo Fisher Scientific) was injected into the retrobulbar sinus (125 μg/g body weight) and circulated overnight. The following day, 1 kDa A488-cadaverine (catalog A30676, Invitrogen) was injected in the other eye (10 μg/g body weight) and circulated 2 hours. Anesthetized mice were perfused as mentioned above, and brains were processed for IF staining. For tracer area quantifications, 6 fields from 2 sections per mouse were analyzed from each malformed region (SS4–6, CP), and 1 field from 1 section per mouse from each non-malformed region (AI, ORB, MO1–3, VIS, SS1–3). The areas were measured as explained in *Quantifications*.

### Microvascular fragment isolation for scRNA-seq and qPCR.

The cerebra were collected and the microvascular fragments were isolated as previously described (24) with minor modifications before 10x Genomics scRNA-seq; between primary and secondary dissociation steps, samples were kept at 4°C.

### qPCR.

mRNA from microvascular fragments was extracted using the RNeasy microprep kit (catalog 74034, QIAGEN). cDNA was synthesized with iScript Reverse Transcription Supermix (catalog 1708840, Bio-Rad), and TaqMan assays were performed from 80 ng of cDNA per mouse. *Gapdh* was used as a housekeeping gene. Specific probes used are listed in Supplemental Table 1.

### ScRNA-seq.

Single-cell suspensions (viability >92% assessed with LUNA-FL Dual Fluorescence cell counter, Logos Biosystems) were processed using a Next GEM Single Cell 3′ Reagent kit v3.1 with dual index (10x Genomics). cDNA libraries from about 5,000 cells per sample were subsequently sequenced on a NextSeq 2000 sequencer (Illumina) with P3 100-cycle flow cell.

Data were demultiplexed and aligned to the mouse genome (GRm38/mm10) using Cell Ranger v6.1.2 software (10x Genomics) and loaded into R software (v4.1.1, R Core Team) using the Seurat R package (v4.3.0) (42, 43). Low-quality transcriptomes (detected genes ≤500, detected mitochondrial genes ≥10%) were removed. Seurat pipeline was used to compute principal component analysis (PCA) (*n* = 50), clusters (resolution = 0.2), and uniform manifold approximation and projection (UMAP) distribution. Before in-depth analysis, generally accepted markers to identify clusters of ECs were used. Thereafter, 14,120 EC transcriptomes were selected for downstream analysis (PCA, *n* = 50; clusters, resolution = 0.4 or 0.6). Cluster-enriched marker genes were identified using the FindAllMarkers() function (min.pct = 0.5, logfc.threshold = 0.5). DEGs in cluster 4 were identified with the FindMarkers() function comparing cluster 4 with each other cluster separately, with an equal number of cells selected from both groups using the sample() function. Model-based analysis of single-cell transcriptomics (MAST) test (MAST R package version 1.20.0) was used for calculation of adjusted *P* values. Genes from each comparison with average log_2_ fold change ≥ 0.5 *o*r ≤ –0.5 and adjusted *P* value ≤ 0.05 were selected, and common genes were identified using VennDiagram (R package v1.7.3). To identify DEGs between clusters 4i and 4ii, 5 repeated analyses with an equal number of cells selected from both groups were performed, and common DEGs were identified as described above.

Seurat DimPlot(), FeaturePlot(), and VlnPlot() functions were used for data visualization. Seurat DoHeatmap() or the pheatmap() function of the Pretty Heatmaps R package (v1.0.12) was used to create heatmaps. Fractions of cells per cluster were shown as bar plots using the ggplot2 R package, with normalized values, calculated from the overall cell number per genotype per sample.

GO analysis was performed using the clusterProfiler R package (v4.2.2) (44). Biological process sub-ontology terms were probed, using the enrichGO() function with all expressed genes of the EC dataset as reference (universe), minGSSize = 10, masGSSize = 500, pAdjustMethod = Benjamini-Hochberg, and p/qvalueCutoff = 0.05. Then, the simplify() function was used (cutoff = 0.7), and the top 10 terms were visualized using the dotplot() function from the clusterProfiler package.

Bar plots in the online database were obtained from first raw counts per cell normalized to 50,000 counts library size. Thereafter, cells from each cluster were divided by their genotype, and SPIN algorithm (/backspin -I input.cef -o output.cef -f 1000 -b both) (45) was applied to order cells according to their gene expression patterns (genes with ≥100 total counts). The SPIN-ordered cells were then recombined per cluster and visualized using the barplot() R function.

### ANGPT2 spatiotemporal expression analysis.

Brain cerebral cortex, hippocampus, and basal ganglia from C57BL6/J mice at P1, P15, P20, P30, and P60 (1–3 fields per section from 2 sections per mouse) were stained for ANGPT2 and PECAM1. ERG^+^ cells and ANGPT2^+^ERG^+^ cells were manually counted with Fiji.

### Statistics.

The data are shown as mean ± SD. To study normality, Shapiro-Wilk and Kolmogorov-Smirnov tests were used. Normally distributed 2-group comparisons were tested for significance using the 2-tailed, unpaired Student’s *t* test with Welch’s correction. *P* ≤ 0.05 was considered statistically significant for all tests. Normally distributed multiple comparisons with 1 independent variable were tested for significance using the ordinary 1-way ANOVA with α = 0.05 and Tukey’s post hoc method to compare every mean with every other mean. Not normally distributed multiple comparisons were tested for significance using the Kruskal-Wallis test with α = 0.05 and Dunn’s post hoc method to compare the mean ranks between groups. Normally distributed multiple comparisons with multiple independent variables were tested for significance using the 2-way ANOVA with α = 0.05 and Tukey’s post hoc method to compare every mean with every other mean. No prior power calculations were performed.

### Study approval.

All animal experiment protocols were approved by the Uppsala Ethical Committee on Animal Research (5.8.18-03029/2020, 5.8.18-16497.2024) and carried out in accordance with the Committee’s guidelines.

### Data availability.

Data are available in public repositories (Gene Expression Omnibus, accession number GSE317102; and in searchable database: https://andaloussimaelab.org/publications/ang2/database.html); in the Supporting Data Values file; or upon request.

## Author contributions

MAM conceptualized the study. WL, EVL, LM, LH, PB, DES, FS, CF, JW, MV, and AN performed investigation. MAM, EVL, and WL wrote the original draft of the manuscript. MAM, EVL, WL, LM, and MJ reviewed and edited the manuscript. EVL, WL, and FS performed visualization. WL performed quantification. MAM supervised the study.

## Funding support

The following entities provided financial support.

- Swedish Research Council (2023-02655 to MAM).
- Leducq Foundation (23CVD02 to MAM).
- European Foundation for the Study of Diabetes (EFSD-BI-2023 to MJ).
- Swedish Kidney Foundation (F2021-0061, F2022-0046, and F2023-0064 to MJ).
- National Natural Science Foundation of China (82401605 to JW).
- China Scholarship Council (CSC 202006210088 to WL).

## Supplementary Material

Supplemental data

Supplemental table 2

Supplemental table 3

Supplemental table 4

Supplemental table 5

Supplemental table 6

Supplemental table 7

Supplemental table 8

Supplemental table 9

Supplemental video 1

Supplemental video 2

Supporting data values

## Version 1 02/19/2026

In-Press Preview

## Version 2 04/08/2026

Electronic publication

## References

[1] Profaci CP (2020). The blood-brain barrier in health and disease: important unanswered questions. J Exp Med.

[2] Saharinen P (2017). Therapeutic targeting of the angiopoietin-TIE pathway. Nat Rev Drug Discov.

[3] Jeansson M (2011). Angiopoietin-1 is essential in mouse vasculature during development and in response to injury. J Clin Invest.

[4] Suri C (1996). Requisite role of angiopoietin-1, a ligand for the TIE2 receptor, during embryonic angiogenesis. Cell.

[5] Gale NW (2002). Angiopoietin-2 is required for postnatal angiogenesis and lymphatic patterning, and only the latter role is rescued by Angiopoietin-1. Dev Cell.

[6] Hackett SF (2000). Angiopoietin 2 expression in the retina: upregulation during physiologic and pathologic neovascularization. J Cell Physiol.

[7] Hackett SF (2002). Angiopoietin-2 plays an important role in retinal angiogenesis. J Cell Physiol.

[8] Felcht M (2012). Angiopoietin-2 differentially regulates angiogenesis through TIE2 and integrin signaling. J Clin Invest.

[9] Kim M (2016). Opposing actions of angiopoietin-2 on Tie2 signaling and FOXO1 activation. J Clin Invest.

[10] Korhonen EA (2016). Tie1 controls angiopoietin function in vascular remodeling and inflammation. J Clin Invest.

[11] Mäe MA (2021). Single-cell analysis of blood-brain barrier response to pericyte loss. Circ Res.

[12] Vazquez-Liebanas E (2024). Mosaic deletion of claudin-5 reveals rapid non-cell-autonomous consequences of blood-brain barrier leakage. Cell Rep.

[13] Vazquez-Liebanas E (2022). Adult-induced genetic ablation distinguishes PDGFB roles in blood-brain barrier maintenance and development. J Cereb Blood Flow Metab.

[14] Zhou X (2023). ANG2 blockade diminishes proangiogenic cerebrovascular defects associated with models of hereditary hemorrhagic telangiectasia. Arterioscler Thromb Vasc Biol.

[15] Xie Y (2021). Key molecular alterations in endothelial cells in human glioblastoma uncovered through single-cell RNA sequencing. JCI Insight.

[16] Xie Y (2024). Single-cell dissection of the human blood-brain barrier and glioma blood-tumor barrier. Neuron.

[17] Callegari K (2023). Molecular profiling of the stroke-induced alterations in the cerebral microvasculature reveals promising therapeutic candidates. Proc Natl Acad Sci U S A.

[18] Armulik A (2010). Pericytes regulate the blood-brain barrier. Nature.

[19] Vanlandewijck M (2018). A molecular atlas of cell types and zonation in the brain vasculature. Nature.

[20] Barclay KM (2024). An inducible genetic tool to track and manipulate specific microglial states reveals their plasticity and roles in remyelination. Immunity.

[21] Siret C (2022). Deciphering the heterogeneity of the Lyve1^+^ perivascular macrophages in the mouse brain. Nat Commun.

[22] Butovsky O, Weiner HL (2018). Microglial signatures and their role in health and disease. Nat Rev Neurosci.

[23] Paolicelli RC (2022). Microglia states and nomenclature: a field at its crossroads. Neuron.

[24] Bjornholm KD (2023). A robust and efficient microvascular isolation method for multimodal characterization of the mouse brain vasculature. Cell Rep Methods.

[25] Thomson BR (2014). A lymphatic defect causes ocular hypertension and glaucoma in mice. J Clin Invest.

[26] del Toro R (2010). Identification and functional analysis of endothelial tip cell-enriched genes. Blood.

[27] Strasser GA (2010). Microarray analysis of retinal endothelial tip cells identifies CXCR4 as a mediator of tip cell morphology and branching. Blood.

[28] Lupu IE (2025). Direct specification of lymphatic endothelium from mesenchymal progenitors. Nat Cardiovasc Res.

[29] Souma T (2018). Context-dependent functions of angiopoietin 2 are determined by the endothelial phosphatase VEPTP. Proc Natl Acad Sci U S A.

[30] Bae H (2020). Angiopoietin-2-integrin α5β1 signaling enhances vascular fatty acid transport and prevents ectopic lipid-induced insulin resistance. Nat Commun.

[31] Daly C (2004). Angiopoietin-1 modulates endothelial cell function and gene expression via the transcription factor FKHR (FOXO1). Genes Dev.

[32] Kim I (2000). Angiopoietin-1 regulates endothelial cell survival through the phosphatidylinositol 3′-kinase/Akt signal transduction pathway. Circ Res.

[33] Vanlandewijck M (2015). Functional characterization of germline mutations in PDGFB and PDGFRB in primary familial brain calcification. PLoS One.

[34] Phoenix TN (2016). Medulloblastoma genotype dictates blood brain barrier phenotype. Cancer Cell.

[35] Sabbagh MF, Nathans J (2020). A genome-wide view of the de-differentiation of central nervous system endothelial cells in culture. Elife.

[36] Gerhardt H (2003). VEGF guides angiogenic sprouting utilizing endothelial tip cell filopodia. J Cell Biol.

[37] Simons M (2016). Mechanisms and regulation of endothelial VEGF receptor signalling. Nat Rev Mol Cell Biol.

[38] Gelfand MV (2014). Neuropilin-1 functions as a VEGFR2 co-receptor to guide developmental angiogenesis independent of ligand binding. Elife.

[39] Lallemand Y (1998). Maternally expressed PGK-Cre transgene as a tool for early and uniform activation of the Cre site-specific recombinase. Transgenic Res.

[40] Percie du Sert N (2020). Reporting animal research: explanation and elaboration for the ARRIVE guidelines 2.0. PLoS Biol.

[41] Liu L (2022). F-CUBIC: a rapid optical clearing method optimized by quantitative evaluation. Biomed Opt Express.

[42] Satija R (2015). Spatial reconstruction of single-cell gene expression data. Nat Biotechnol.

[43] Butler A (2018). Integrating single-cell transcriptomic data across different conditions, technologies, and species. Nat Biotechnol.

[44] Wu T (2021). clusterProfiler 4.0: a universal enrichment tool for interpreting omics data. Innovation (Camb).

[45] Tsafrir D (2005). Sorting points into neighborhoods (SPIN): data analysis and visualization by ordering distance matrices. Bioinformatics.

