# Supplementary material for: Loss of angiopoietin-2 leads to region-specific brain malformations and blood-brain barrier leakage
Source: JCI Insight. 2026 Feb 19;11(7):e198256. doi: 10.1172/jci.insight.198256 (PMC13134733; doi:10.1172/jci.insight.198256)
Supplement: Supplemental data [file jciinsight-11-198256-s128.pdf]

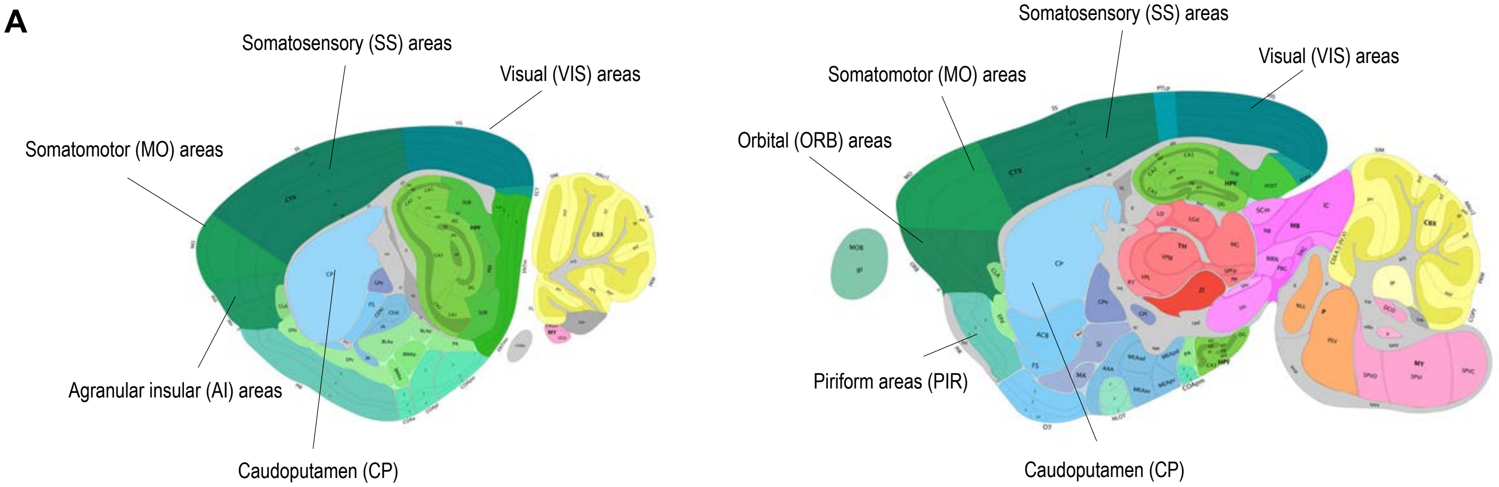

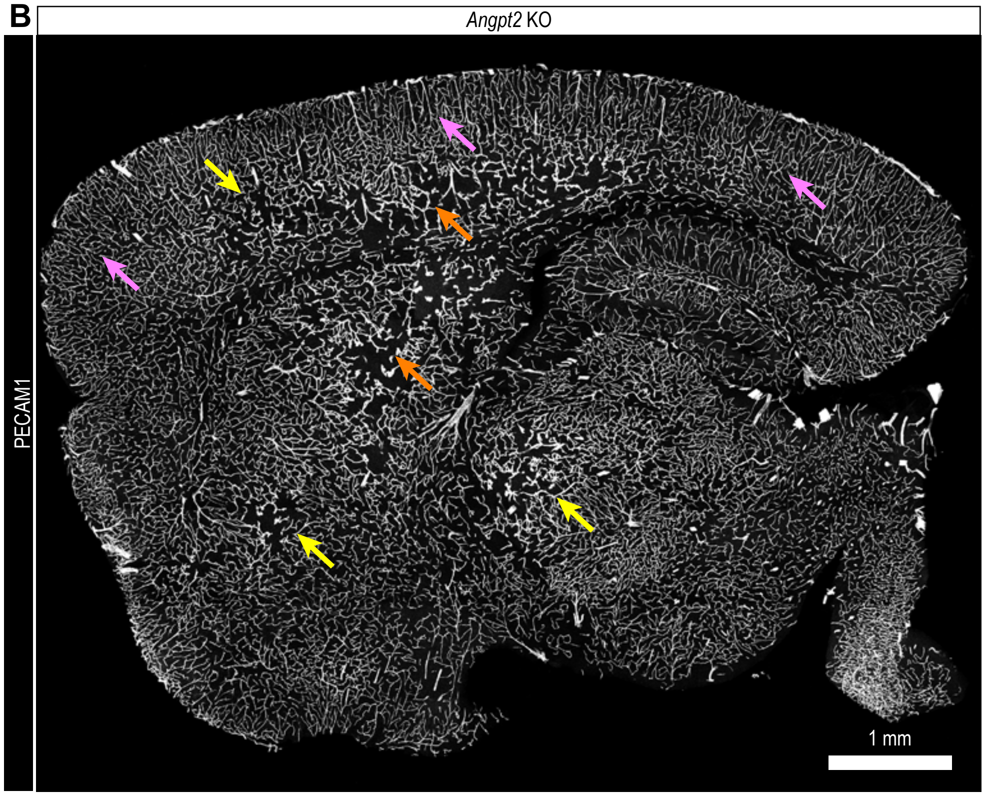

Supplemental Figure 1. A) Anatomical regions of interest highlighted on sagittal sections from Allen Mouse Brain Atlas depicting lateral (left) and medial section (right) (mouse.brain-map.org/experiment/show/100042147). **B)** Example of rare malformations found outside the SS and CP in *Angpt2* KO brains. PECAM1 immunostaining is shown in white. Orange arrows mark malformed vessels in the consistently abnormal areas (SS4-6 and CP). Yellow arrows mark malformed vessels in the non-consistantly abnormal areas (MO, the thalamus and the nucleus accumbens). Pink arrows mark the regions with consistently normal vascular morphology (SS cortex layer 1, VIS and rostral part of MO cortex).

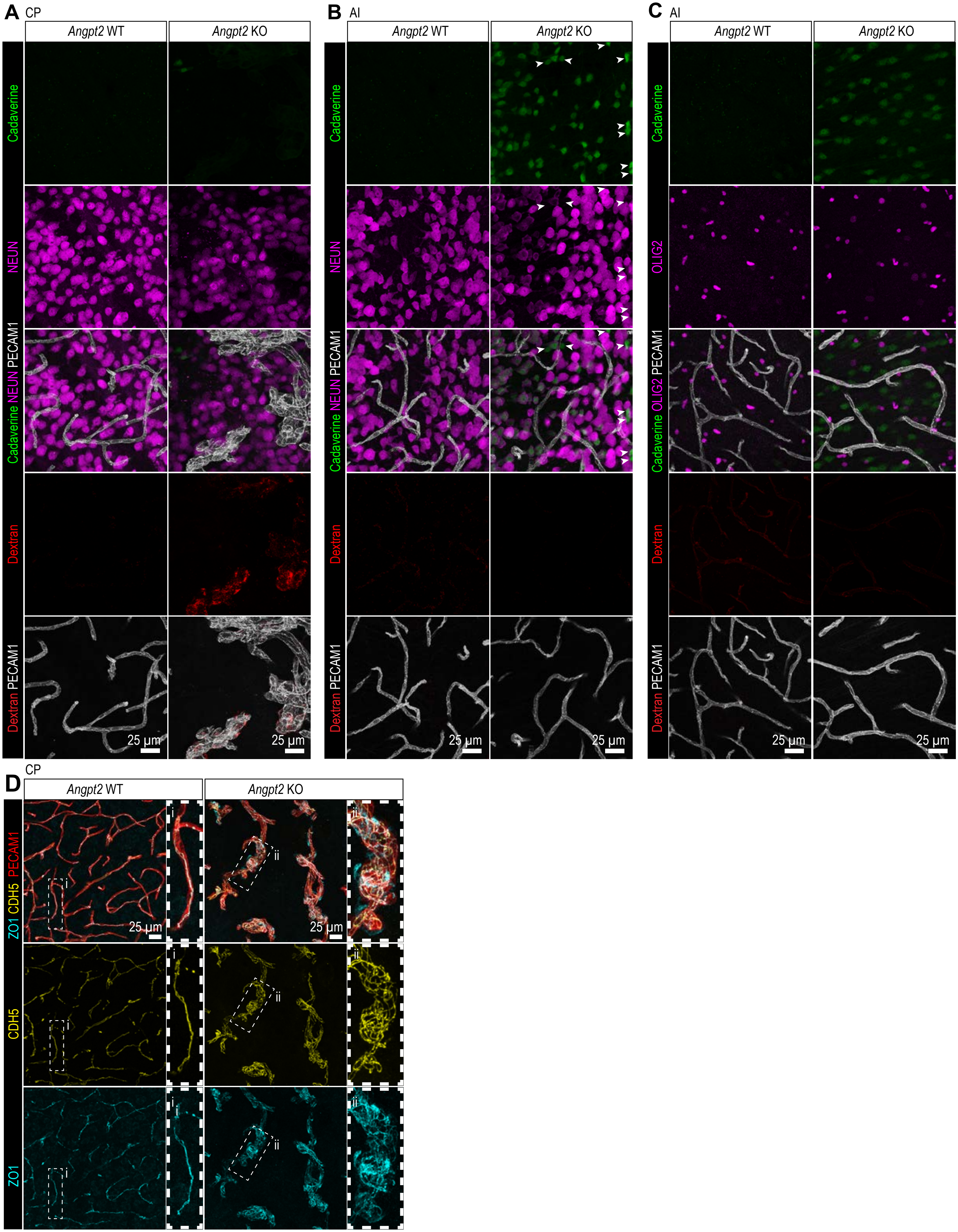
A CP
Angpt2 WT
Angpt2 KO
Cadaverine
NEUN
Cadaverine NEUN PECAM1
Dextran
Dextran PECAM1
25 µm
B AI
Angpt2 WT
Angpt2 KO
Cadaverine
NEUN
Cadaverine NEUN PECAM1
Dextran
Dextran PECAM1
25 µm
C AI
Angpt2 WT
Angpt2 KO
Cadaverine
OLIG2
Cadaverine OLIG2 PECAM1
Dextran
Dextran PECAM1
25 µm
D CP
Angpt2 WT
Angpt2 KO
ZO1 CDH5 PECAM1
CDH5
ZO1
i
ii
25 µm

Supplemental Figure 2. BBB permeability and endothelial junction characterization in constitutive *Angpt2* KO brains. **A, B)** Representative images of the immunostaining of PECAM1 (grey), NEUN (magenta) on 70 kDa TMR-dextran (red) and 1 kDa A488-cadaverine (green) injected mice in *Angpt2* WT and KO brains (n=4). CP (A), AI (B). **C)** Representative images of the immunostaining of PECAM1 (grey) and OLIG2 (magenta) on 70 kDa TMR-dextran (red) and 1 kDa A488-cadaverine (green) injected mice in AI of *Angpt2* WT and KO brains (n=4). **D)** Immunostaining of PECAM1 (red), ZO-1 (cyan) and CDH5 (yellow) in CP of *Angpt2* WT and KO brains (*Angpt2* WT n=5; *Angpt2* KO n=6). Insets show higher magnification of malformed blood vessel in *Angpt2* KO and normal blood vessel in *Angpt2* WT.

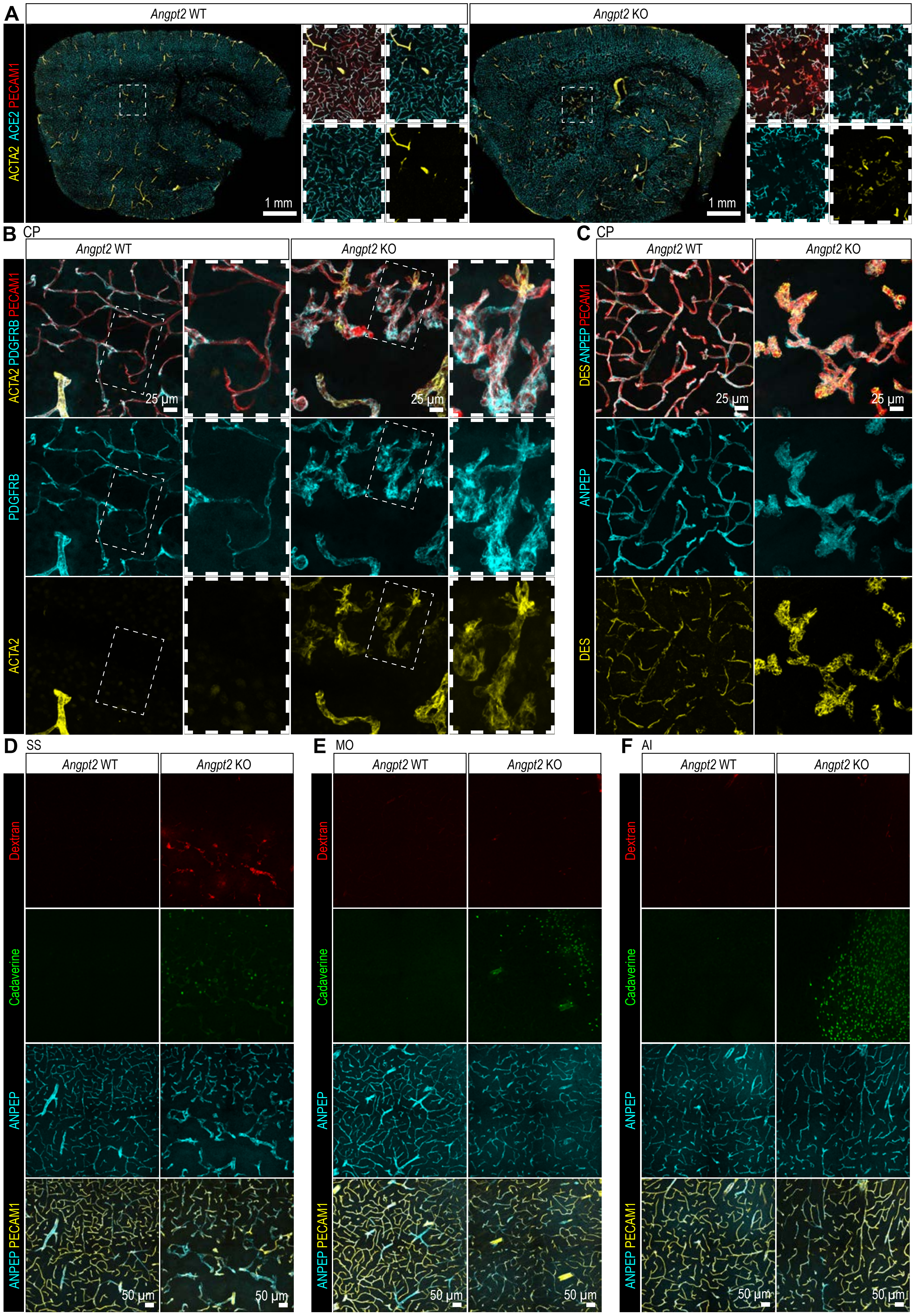

A
Angpt2 WT
Angpt2 KO
ACTA2 ACE2 PECAM1
1 mm
1 mm
B CP
Angpt2 WT
Angpt2 KO
ACTA2 PDGFRB PECAM1
PDGFRB
ACTA2
25 µm
25 µm
C CP
Angpt2 WT
Angpt2 KO
DES ANPEP PECAM1
ANPEP
DES
25 µm
25 µm
D SS
E MO
F AI
Angpt2 WT
Angpt2 KO
Dextran
Cadaverine
ANPEP
ANPEP PECAM1
50 µm

Supplemental Figure 3. Brain perivascular cells phenotypes in adult *Angpt2* KO brains. **A)** Representative tile scans of ACTA2 (yellow) and ACE2 (cyan) immunostainings of sagittal sections from *Angpt2* WT and KO brains (*Angpt2* WT n=5; *Angpt2* KO n=6). **B) I**mmunostaining of ACTA2 (yellow), PDGFRB (cyan) and the endothelial marker PECAM1 (red) from CP of *Angpt2* WT and KO brains (*Angpt2* WT n=5; *Angpt2* KO n=6). **C)** Representative images of the immunostaining of ANPEP (cyan), DESMIN (yellow) and PECAM1 (red) from CP of *Angpt2* WT and KO brains (*Angpt2* WT n=5; *Angpt2* KO n=6). **D-F)** Representative images of the immunostaining of ANPEP (cyan) and PECAM1 (yellow) on 70 kDa TMR-dextran (red) and 1 kDa A488-cadaverine (green) injected brains from *Angpt2* WT and KO brains (n=4). SS (D), MO (E) and AI (F).

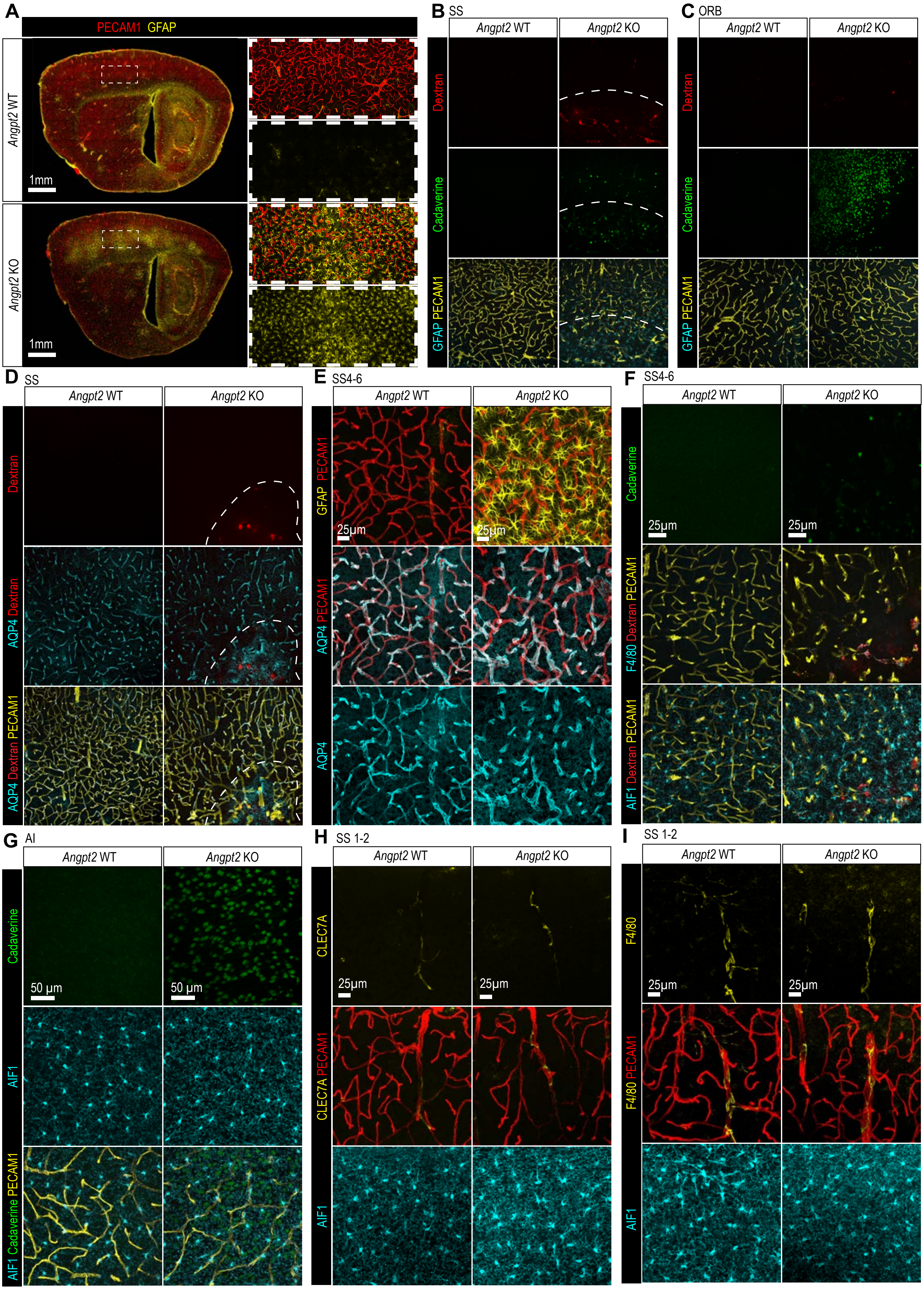
A
PECAM1 GFAP
Angpt2 WT
Angpt2 KO
1mm
1mm
B SS
Angpt2 WT
Angpt2 KO
Dextran
Cadaverine
GFAP PECAM1
C ORB
Angpt2 WT
Angpt2 KO
Dextran
Cadaverine
GFAP PECAM1
D SS
Angpt2 WT
Angpt2 KO
Dextran
AQP4 Dextran
AQP4 Dextran PECAM1
E SS4-6
Angpt2 WT
Angpt2 KO
GFAP PECAM1
25µm
25µm
AQP4 PECAM1
AQP4
F SS4-6
Angpt2 WT
Angpt2 KO
Cadaverine
25µm
25µm
F4/80 Dextran PECAM1
AIF1 Dextran PECAM1
G AI
Angpt2 WT
Angpt2 KO
Cadaverine
50 µm
50 µm
AIF1
AIF1 Cadaverine PECAM1
H SS 1-2
Angpt2 WT
Angpt2 KO
CLEC7A
25µm
25µm
CLEC7A PECAM1
AIF1
I SS 1-2
Angpt2 WT
Angpt2 KO
F4/80
25µm
25µm
F4/80 PECAM1
AIF1

Supplemental Figure 4. Glial phenotypes in *Angpt2* KO brains. **A)** Representative tile scans of GFAP (yellow) and PECAM1 (red) immunostainings on sagittal lateral sections of adult *Angpt2* WT and KO brains (*Angpt2* WT n = 9; *Angpt2* KO n = 10). Insets show the magnifications of astrocytes in *Angpt2* KO WT brains. **B, C)** Representative images of the immunostaining of GFAP (cyan) and PECAM1 (yellow) in SS (B) and ORB (C) of adult *Angpt2* WT and KO brains injected with 1 kDa A488-cadaverine and 70 kDa TMR-dextran (n = 4). **D)** Representative images of the immunostainings of the astrocyte end-feet marker AQP4 (cyan), the endothelial marker PECAM1 (yellow) and 70 kDa TMR-dextran (red) in SS area visualizing both the malformed and surrounding non-malformed vasculature from *Angpt2* KO brains and corresponding region in the WT brain (n = 4). **E)** Representative images of the immunostaining of the astrocyte marker GFAP (yellow), the astrocyte end-feet marker AQP4 (cyan) and the endothelial marker PECAM1 (red) on SS layer 4-6 from *Angpt2* WT and KO brains (n = 4). **F)** Representative images of the immunostainings of the microglial marker AIF1 (cyan), perivascular macrophage maker F4/80 (cyan), the endothelial marker PECAM1 (yellow), 70 kDa TMR-dextran (red) and 1 kDa A488-cadaverine (green) in SS4-6 from *Angpt2* WT and KO brains (n = 4). **G)** Representative images of the immunostainings of AIF1 (cyan) and PECAM1 (yellow) with 1 kDa A488-cadaverine leakage in AI (n = 4). **H)** Representative images of the immunostaining of CLEC7A (yellow), AIF1 (cyan) and PECAM1 (red) in SS1-2 from *Angpt2* WT and KO brains (*Angpt2* WT n = 5; *Angpt2* KO n = 6). **I)** Representative images of the immunostaining of F4/80 (yellow), PECAM1 (red), and AIF1 (cyan) in SS1-2 of adult *Angpt2* WT and KO brains (n = 4).

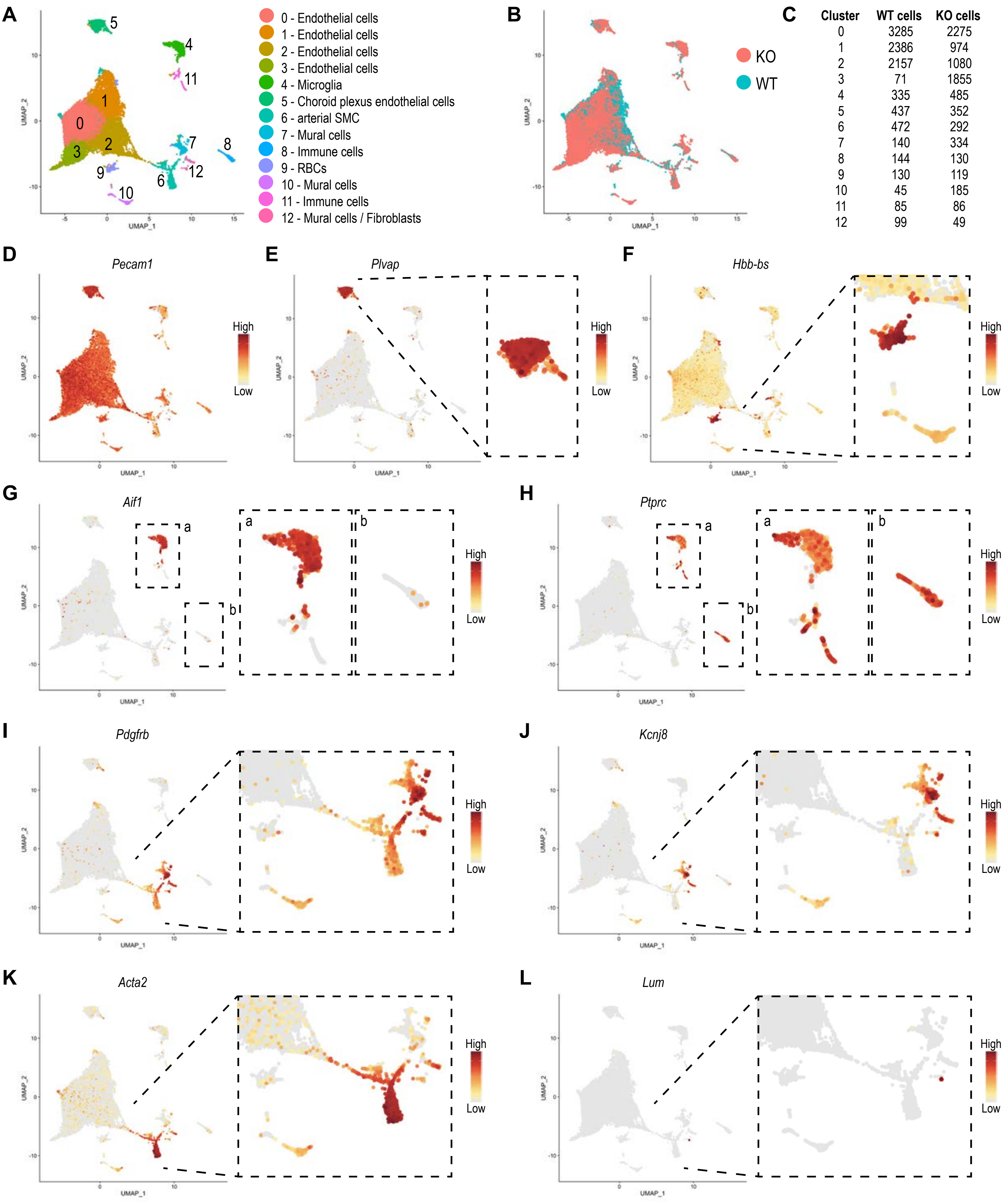

Cluster	WT cells	KO cells
0	3285	2275
1	2386	974
2	2157	1080
3	71	1855
4	335	485
5	437	352
6	472	292
7	140	334
8	144	130
9	130	119
10	45	185
11	85	86
12	99	49

Supplemental Figure 5. ScRNAseq analysis of isolated microvascular cell clustering, marker expression, and differentially expressed genes in *Angpt2* WT and KO mice. **A)** Uniform Manifold Approximation and Projection (UMAP) of vascular cell dataset, clustered into thirteen clusters (0–12). **B)** UMAPs illustrating the distribution of cells from *Angpt2* WT and KO samples. **C)** Total cell number in each cluster per genotype. **D-L** Marker gene expression: **D)** pan-endothelial marker *Pecam1*, **E)** fenestrated endothelial marker *Plvap*, **F)** erythrocyte marker *Hbb-bs*, **G)** microglial marker *Aif*1, **H)** immune cell marker *Ptprc*, **I-K)** mural cell markers *Pdgfrb, Kcnj8, Acta2*, and **L)** fibroblast marker *Lum*.

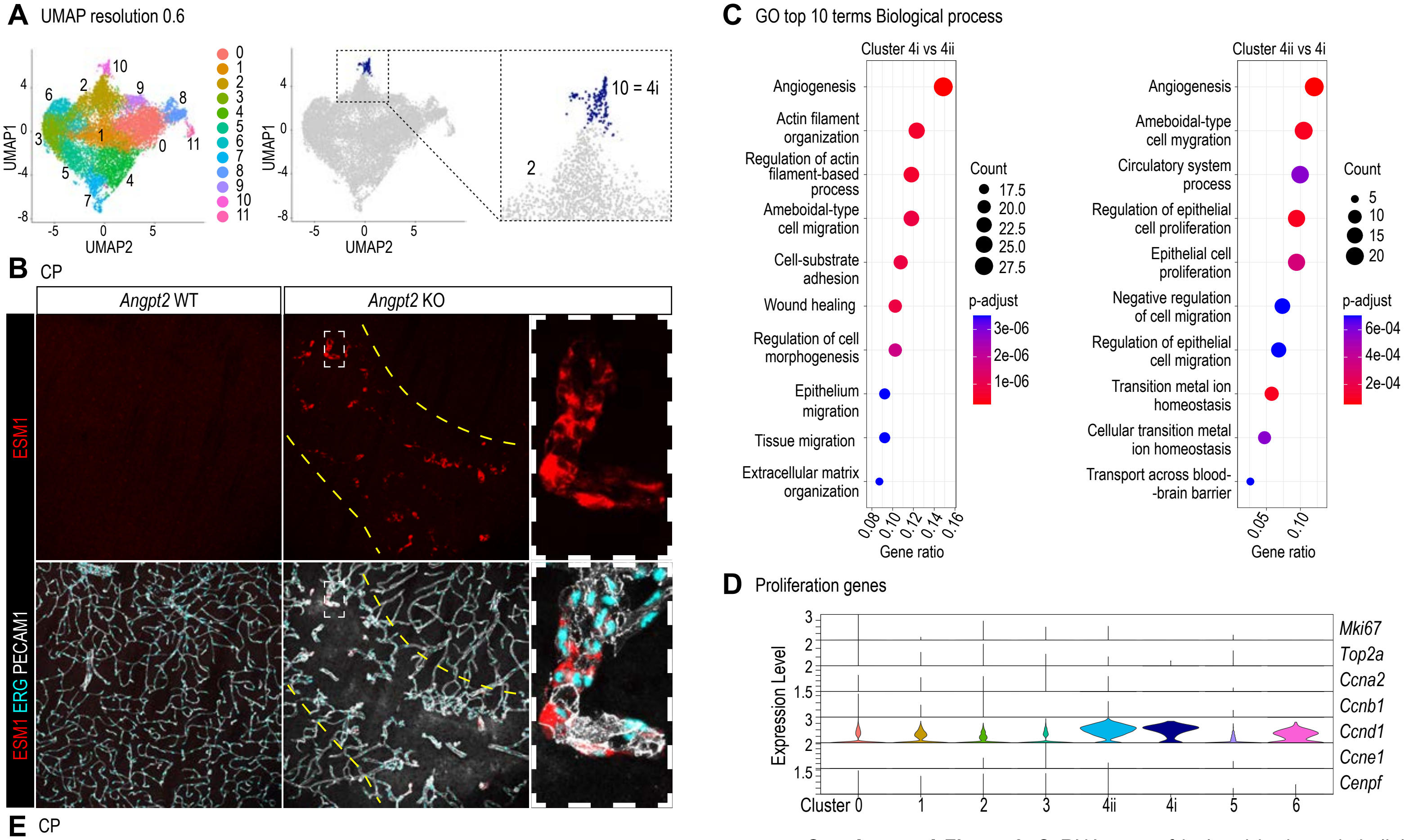

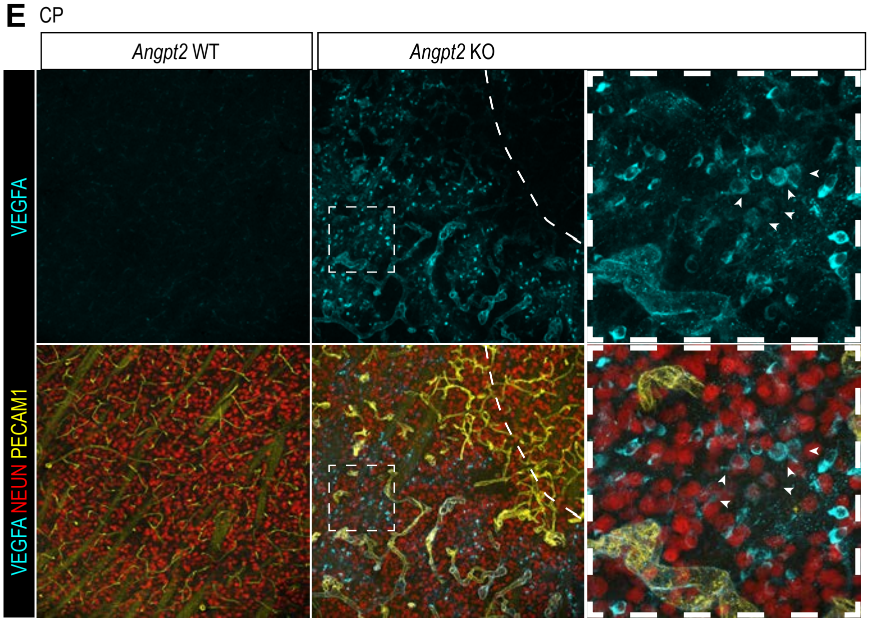

Supplemental Figure 6. ScRNA-seq of isolated brain endothelial cells from *Angpt2* KO and WT brains. **A)** UMAP with resolution 0.6 of the endothelial cells clustered in 12 clusters (0-11). Cluster 10 is incorporated as "4i" to the clustering with resolution 0.4 in Figure 6 and 7. **B)** Representative images of the immunostainings of ESM1 (red), ERG (cyan) and PECAM1 (grey) in the CP of Angpt2 WT and KO brains (n=3). Yellow dashed line marks the boundary between malformed and non-malformed areas. Insets show the filopodia-negative ECs expressing ESM1. **C)** Gene Ontology (GO) biological process analysis showing the top 10 terms of cluster 4i upregulated genes compared with 4ii and vice versa. **D)** Violin plots showing the expression of proliferation markers throughout the 8 clusters. Markers were picked from Vanlandewijck, He et al., reference 33. **E)** Representative images of the immunostainings of VEGFA (cyan), PECAM1 (yellow), and NEUN (red) in the CP of *Angpt2* WT and KO brains (n=3). Dashed line marks the boundary between malformed and non-malformed vasculature. Inset shows a magnification of a region with malformed vasculature and arrowheads point at NEUN/VEGFA positive neurons.

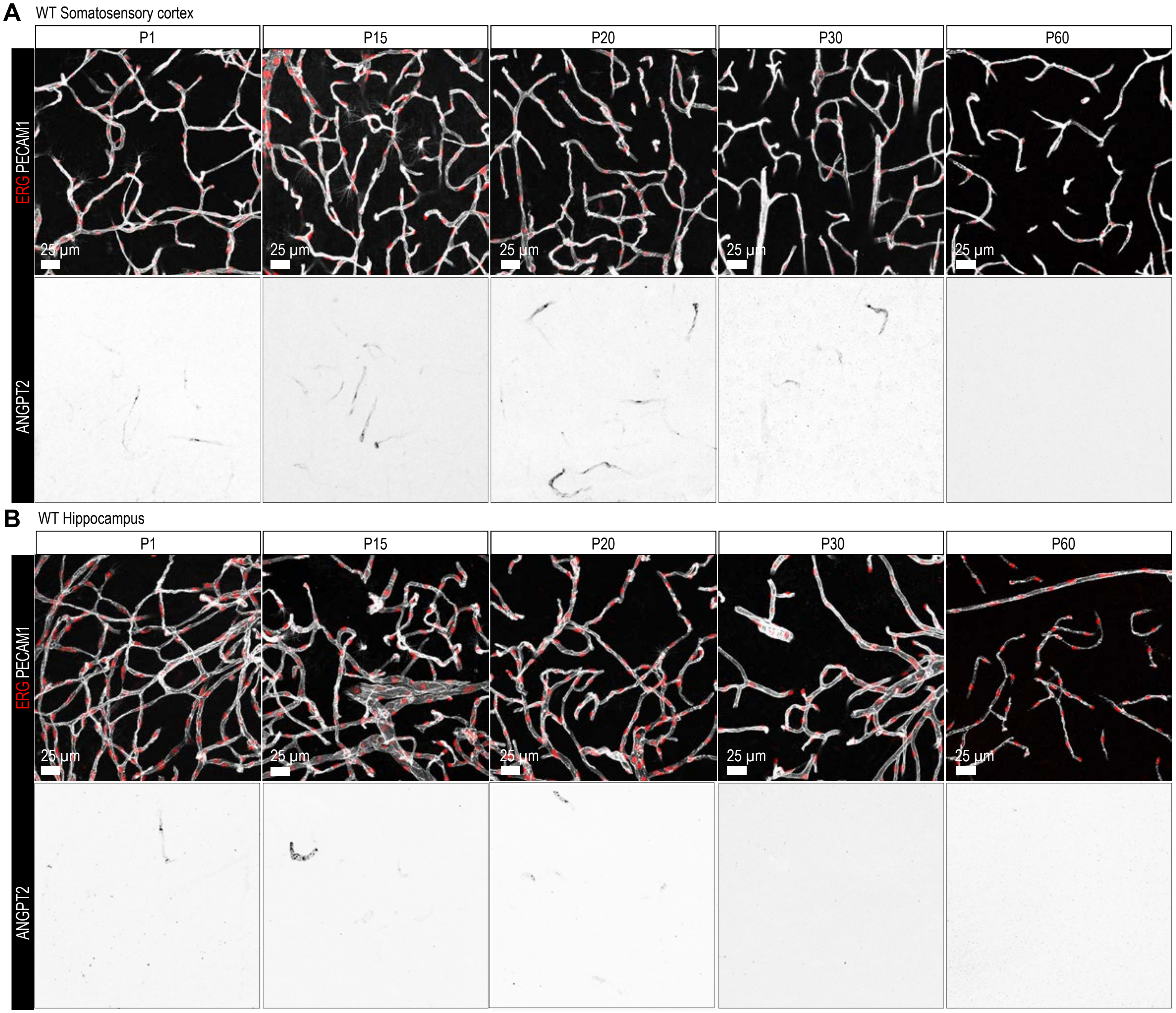

Supplemental Figure 7. Spatiotemporal ANGPT2 expression analysis in C57Bl6/J postnatal brains at P1-60 in SS (**A**) and hippocampus (**B**). Representative images of immunostainings of ERG (red), PECAM1 (grey), ANGPT2 (black) (n=3).

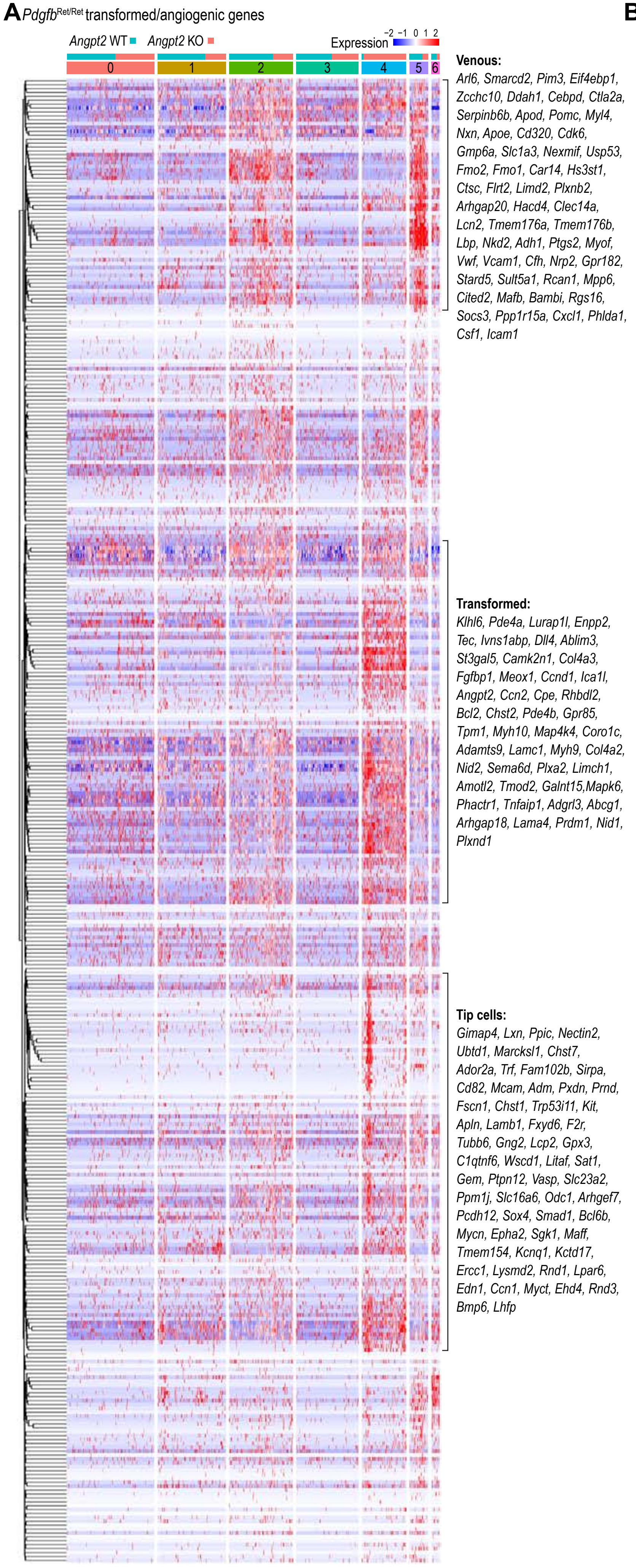

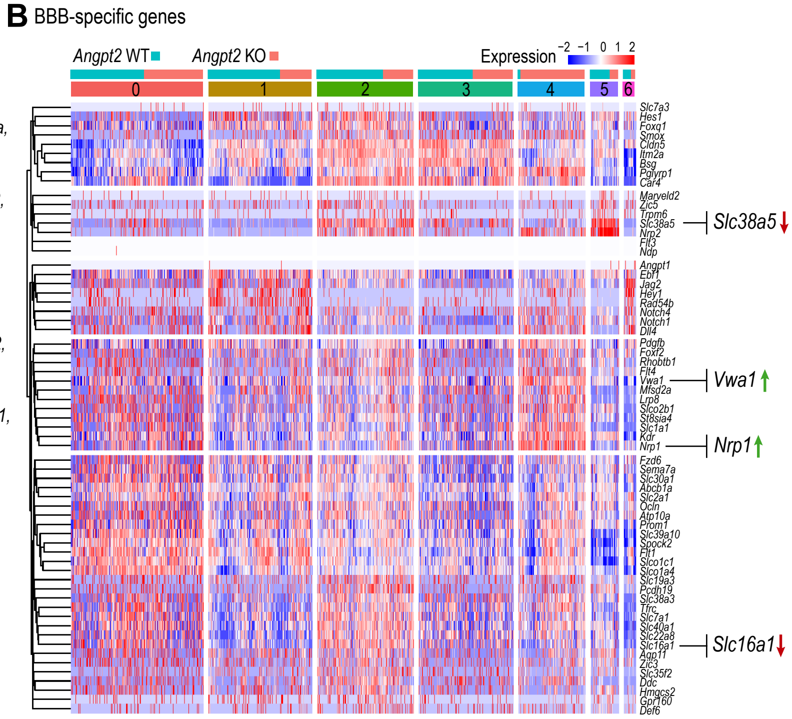

Supplemental Figure 8. Transcriptomic comparison of malformed brain endothelial cells. **A)** Heatmap showing the expression of *Pdgfb*$^{Ret/Ret}$ transformed/angiogenic genes in the *Angpt2* WT and KO endothelial cell clusters 0-6. The selection of *Pdgfb*$^{Ret/Ret}$ transformed/angiogenic genes was based on Mäe et al., reference 11. **B)** Heatmap of expression of BBB-specific genes in clusters 0-6. Selection of genes was based on Phoenix et al, Sabbagh et al., references 34, 35. Red arrows pointing down refer to the genes that were significantly downregulated in cluster 4 compared to the rest of the clusters and green arrows pointing up mean the genes that were significantly upregulated.
